# Determinants of Influenza Mortality Trends: Age-Period-Cohort Analysis of Influenza Mortality in the United States, 1959–2016

**DOI:** 10.1007/s13524-019-00809-y

**Published:** 2019-09-09

**Authors:** Enrique Acosta, Stacey A. Hallman, Lisa Y. Dillon, Nadine Ouellette, Robert Bourbeau, D. Ann Herring, Kris Inwood, David J. D. Earn, Joaquin Madrenas, Matthew S. Miller, Alain Gagnon

**Affiliations:** 1 Département de Démographie, Université de Montréal, C.P. 6128, succursale Centre-ville, Montréal, QC H3C 3J7 Canada; 2 Max Planck Institute for Demographic Research, Rostock, Germany; 3 Demography Division, Statistics Canada, Ottawa, Canada; 4 Department of Anthropology, McMaster University, Hamilton, Canada; 5 Department of History, University of Guelph, Guelph, Canada; 6 Department of Mathematics and Statistics, McMaster University, Hamilton, Canada; 7 Michael G. DeGroote Institute for Infectious Diseases Research, McMaster University, Hamilton, Canada; 8 Los Angeles Biomedical Research Institute, Harbor-UCLA Medical Center, Torrance, CA USA; 9 Department of Biochemistry and Biomedical Sciences, McMaster, Hamilton, Canada; 10 McMaster Immunology Research Centre, McMaster University, Hamilton, Canada; 11 Public Health Research Institute (IRSPUM), Université de Montréal, Montreal, Canada

**Keywords:** Influenza mortality, Antigenic imprinting, Cohort morbidity phenotype, Lexis surfaces, Age-period-cohort analysis

## Abstract

This study examines the roles of age, period, and cohort in influenza mortality trends over the years 1959–2016 in the United States. First, we use Lexis surfaces based on Serfling models to highlight influenza mortality patterns as well as to identify lingering effects of early-life exposure to specific influenza virus subtypes (e.g., H1N1, H3N2). Second, we use age-period-cohort (APC) methods to explore APC linear trends and identify changes in the slope of these trends (contrasts). Our analyses reveal a series of breakpoints where the magnitude and direction of birth cohort trends significantly change, mostly corresponding to years in which important antigenic drifts or shifts took place (i.e., 1947, 1957, 1968, and 1978). Whereas child, youth, and adult influenza mortality appear to be influenced by a combination of cohort- and period-specific factors, reflecting the interaction between the antigenic experience of the population and the evolution of the influenza virus itself, mortality patterns of the elderly appear to be molded by broader cohort factors. The latter would reflect the processes of physiological capital improvement in successive birth cohorts through secular changes in early-life conditions. Antigenic imprinting, cohort morbidity phenotype, and other mechanisms that can generate the observed cohort effects, including the baby boom, are discussed.

**Electronic supplementary material:**

The online version of this article (10.1007/s13524-019-00809-y) contains supplementary material, which is available to authorized users.

## Introduction

At the beginning of the twentieth century, pneumonia and influenza (P&I) were the leading causes of death in the United States (Deaton 2015), and today, they remain the most important causes of death among infectious diseases (Armstrong et al. 1999). The Spanish flu (1918–1920), also known as the “mother of all pandemics” (Taubenberger and Morens 2006), caused more deaths than World War I and killed more people in 24 weeks than AIDS did over a span of 24 years (Barry 2005). The following three influenza pandemics (1957, 1968, and 2009) and the appearance of new subtypes, such as the highly pathogenic avian H5N1 and H7N9 influenza viruses (Haque et al. 2007), demonstrate that influenza remains a significant threat to public health. Population aging makes it likely that casualties will increase (Simonsen et al. 2011), given that about 90 % of all influenza deaths occur among people aged 65 and over (Thompson et al. 2003).

As is true for most infectious diseases, mortality from influenza diminished appreciably during the twentieth century (Armstrong et al. 1999). However, there is still much uncertainty regarding the mechanisms responsible for this reduction. The emphasis has been on monitoring disease and mortality from specific strains from year to year, with indicators of virulence, basic reproductive number (the number of people infected by one index case), or attack rates (the percentage of people infected) broken down by geographic areas and broadly defined age groups (Reichert et al. 2004; Thompson et al. 2010; Thompson et al. 2003). The generalized nature of these investigations has fostered interpretations of change over time almost exclusively in terms of secular or period change, modulated by biological age, with few alternative explanations of time trends. More recently, some investigators have focused on age-specific mortality from influenza during pandemics (Lemaitre et al. 2012; Nguyen and Noymer 2013), while others have analyzed the consequences of early-life exposure to pandemic influenza on health and mortality in general (Almond 2006; Kelly 2009; Mazumder et al. 2010) or on mortality during subsequent influenza pandemics (Gagnon et al. 2013; Hallman 2015; Hallman and Gagnon 2014; Ma et al. 2011; Oeppen and Wilson 2006; Viboud et al. 2010). To our knowledge, only a few studies (see, e.g., Azambuja 2009, 2015; Cohen et al. 2010) have undertaken an analysis of influenza mortality variation over time in an age-period-cohort (APC) framework.

In most previous analyses, information on age was analyzed using five-year (or larger) age groups, allowing for broad distinctions in mortality patterns among infants, children, adolescents, adults, and seniors, but affording few chances to identify cohort effects defined on a yearly basis, as pertaining to cohorts born during a pandemic year. There is also nonnegligible heterogeneity within broadly defined age categories, especially when referring to individuals in a terminal age category as broad as 65+ years. Some studies have shown an important change in influenza mortality risk after age 60, with an 11-fold higher risk for a senior older than 80 compared with persons 65–69 (Simonsen et al. 2005, 2011; Thompson et al. 2009).

The present study examines the roles of age, period, and cohort factors as drivers of influenza mortality change over the years 1959–2016 in the United States. It also addresses the effect of early-life exposure to the different influenza A virus (IAV) subtypes that have circulated over the past decades on later-life mortality in the United States for single-year ages, periods, and cohorts, focusing on both seasonal epidemic and pandemic periods. To this end, we first estimated influenza mortality from death records by single years of age in the United States from 1959 to 2016 using Serfling models based on mortality data (Serfling 1963). Second, we used Surveillance-Serfling models (Thompson et al. 2009) accounting for influenza-like illness incidence between 1997 and 2016 to improve parametrization of the Serfling model and to evaluate the effect of influenza virus subtype on mortality by age. Then we constructed Lexis surfaces from influenza death rates and applied detrended APC models (Carstensen 2007; Clayton and Schifflers 1987; Holford 1991) and the intrinsic estimator model (Fu 2000; Yang et al. 2004) to explore period and cohort effects on mortality variation. We also estimated *contrasts*, proposed by Tarone and Chu (1996), to identify statistically significant changes in mortality risk along birth cohort trends. We interpret our results mainly in light of the *antigenic imprinting hypothesis* (Davenport et al. 1953; Ma et al. 2011) and the *cohort morbidity phenotype hypothesis* (Finch and Crimmins 2004), described in the next section.

## Age-Period-Cohort Effects on Influenza Mortality

Susceptibility to infection and mortality from influenza chiefly depends on virus-host interaction factors and on the evolution of the virus itself (Thompson et al. 2003). Because the immune response generated against a given strain of the IAV is not fully cross-protective, the virus can evade the host’s immunity from one season to the next by accumulating mutations that change its antigenicity. This process—antigenic *drift—*is differentiated from the appearance of a novel IAV by reassortment of the HA and NA surface proteins of IAV—antigenic *shift*—which can lead to pandemics (Nelson and Holmes 2007).

Whereas typical IAV seasonal outbreaks most seriously affect the elderly (Thompson et al. 2010), epidemiological analyses of influenza pandemics have revealed a shift of mortality toward younger ages, as was the case during the 1918, 1968, and 2009 pandemics (Nguyen and Noymer 2013; Oeppen and Wilson 2006; Simonsen et al. 1998). During these outbreaks, older individuals often benefited from immunity acquired from previous exposures to virus strains similar to the current pandemic strain, whereas younger adults and children were at higher risk because of a lack of cross-protection from previous infections by similar IAVs. Risk can also be compounded in younger individuals, whose strong immune response to the virus can quickly turn overreactive and dysregulated, leading to immunopathology and organ damage (Kobasa et al. 2007; Loo and Gale 2007; Shanks and Brundage 2012).

The *antigenic imprinting hypothesis* additionally postulates that mortality from influenza depends not only on the virulence of the circulating strain but also on the strain to which a specific cohort was primed (Davenport et al. 1953; Ma et al. 2011; Rajendran et al. 2017). This original strain would indeed keep its senior position in the immune repertoire over successive episodes of infection, with each novel strain taking a more junior position (Henry et al. 2018; Miller et al. 2013). Based on studies showing the variable efficacy of repeated annual influenza vaccination (Smith et al. 1999), protection is expected when the original strain is similar to the circulating strain; however, if the two are very dissimilar, susceptibility to severe outcome may increase (Cobey and Hensley 2017). According to this hypothesis, infection in the first years of life with a H3N8 virus, as was presumably the case for those born during the 1890 Russian IAV pandemic (Worobey et al. 2014), increased the risk of death upon encounter with the doubly heterosubtypic H1N1 virus that was responsible for the Spanish flu pandemic in 1918 (Gagnon et al. 2013; Hallman and Gagnon 2014; Shanks and Brundage 2012). Corroborating this, 50 years later during the 1968 H3N2 Hong Kong flu pandemic, the largest excess mortality was for those aged 50 or a little older (Gagnon et al. 2015). Similarly, a peak in excess mortality during the 2009 H1N1 pandemic was observed at age 52—that is, for those born in 1957—at the time of the H2N2 Asian flu pandemic (Gagnon et al. 2018a).

Hence, whereas mortality at all ages during a given year should reflect the virulence of the circulating strain that year, mortality levels of a specific cohort are expected to reflect the antigenic distance between this strain and the first strain this cohort encountered in early life. The priming of specific cohorts to specific viral strains is thus expected to produce punctual cohort-specific influences, independently of period or cohort trends—that is, longer-term ascending or descending mortality trends that persist over time.

Patterns of influenza mortality may also be interpreted in the light of broader theoretical perspectives, such as Finch and Crimmins’ cohort morbidity phenotype hypothesis (2004), which attributes the vast reductions in later-life mortality from chronic conditions over the last 200 years to the secular reduction in infections during early life. Together, improvements in nutrition and the declining incidence of infectious diseases have been almost continuous since the Industrial Revolution (Floud et al. 2011). Both are believed to have played a salient role in boosting *physiological capital*, an initial health advantage resulting from improved conditions during infancy and early childhood, leading to large increases in life expectancy (Fogel and Costa 1997; Meslé and Vallin 2000).

The cohort morbidity phenotype hypothesis specifically addresses secular changes in mortality from chronic or nontransmissible diseases in old age. Yet, much research has also documented strong comorbidities between chronic diseases and influenza-related mortality for people over age 65 (Plans-Rubió 2007; Reichert et al. 2004; Simonsen et al. 2005). The cohort morbidity perspective provides a rationale to address past reductions in mortality from influenza from a cohort perspective—and more generally from an APC perspective—and not only as the result of secular (period) changes. In other words, improved survival from IAV infections in successive cohorts of elderly could have resulted not only from enhanced responses to infection and to medical treatments but also from delayed onset of comorbid conditions involving influenza and chronic diseases. In this respect, other cohort processes may also shape influenza mortality via these comorbidities or its relationship with mortality in general. For instance, increases in all-cause mortality have also been documented for the baby boomer generation in recent years (Canudas-Romo and Guillot 2015; Rau et al. 2013), and it is possible that what drives these cohort processes also partly drives influenza mortality. Thus, our study also briefly addresses the baby boom as a possible contributor to APC trends in influenza mortality.

## Data and Methods

### Data

Aggregate U.S. death counts by month, single years of age, cause, and sex between January 1959 and December 2016 were obtained from the National Center for Health Statistics (2018). These data cover four successive revisions of the International Statistical Classification of Diseases (from ICD-7 to ICD-10) to classify the deaths. Concordance tables bridged the 7th, 8th, and 9th to the 10th ICD revisions to ensure consistency of definitions for disease categories under study and their comparability over time (Anderson et al. 2001; Klebba and Dolman 1975; Klebba and Scott 1980). Annual counts of population at risk from 1959 to 2016 by single years of age were taken from the Human Mortality Database (2019); monthly counts were estimated through interpolation. Monthly indicators of influenza-like illness (ILI) and percentages of respiratory specimens testing positive for influenza between 1997 and 2016 were estimated from weekly indicators registered in the Centers for Disease Control (CDC) FluView Interactive database (2018). All these data are openly available in the referenced websites. Annual percentages of respiratory specimens testing positive for influenza between 1976 and 1996 were obtained from Thompson et al. (2003).

### Influenza Mortality

Measuring and estimating cause-specific mortality is challenging. Mortality from influenza is no exception (Thompson et al. 2009). On one hand, death records do not contain information from laboratory tests to confirm influenza as the underlying cause of death. Therefore, many deaths recorded as “deaths from influenza” may, in fact, result from morbid events initiated by a disease other than influenza. On the other hand, an influenza infection could trigger a wide spectrum of secondary complications, such as bacterial infections, heart disease, or kidney and diabetes complications, among others (Simonsen et al. 2011), and many deaths primarily due to influenza infections may be wrongly attributed to another cause. Previous analyses of U.S. death certificates confirm that reports of influenza as a standalone cause of death are not to be trusted and should be regrouped first with other causes of death prior its estimation and analysis (Noymer and Nguyen 2013). Given that our purpose is to specifically account for APC effects on influenza mortality, and not to precisely estimate general influenza mortality levels, we estimated the Serfling models based on the restricted P&I cause-of-death category.

### The Serfling Regression Model

**Fig. 1:**
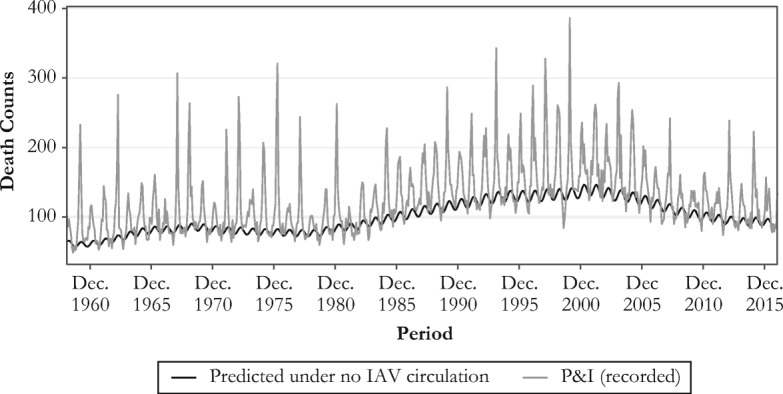
Monthly observed P&I death counts and baseline mortality (without influenza activity) predicted by the Serfling model at age 80, 1959–2016

Serfling models estimate a mortality baseline by fitting death counts of the summer season, during which influenza virus does not circulate widely, while taking into account seasonal and secular mortality trends (Serfling 1963; Thompson et al. 2009). Influenza-related mortality is then estimated for each month as the difference between the observed P&I death counts and the estimated baseline (see Fig. 1). Note that the amplitude of the baseline (and thus the estimated number of deaths) depends on the months chosen to define the summer period. According to our estimates, the best fit was obtained when using a summer period from June to September (details of the Serfling model and the sensitivity tests of alternative summer period definitions are provided in the online appendix).

One important advantage of the Serfling model is that it requires only death counts and populations at risk by month and age. In the present case, it permits the estimation of influenza mortality over a long period (from 1959 to 2016). However, because it relies strongly on seasonal variations, this model may capture unrelated mortality that follows a similar seasonal pattern leading to incoherent estimates, such as a negative number of influenza deaths (Nguyen and Noymer 2013)—hence the interest of the Surveillance-Serfling model described next.

### The Surveillance-Serfling Regression Model

Besides the three components of the Serfling regression model (time trend, seasonality, and population at risk), the Surveillance-Serfling model can also take into account influenza morbidity indicators—such as ILI and other viral surveillance data—which may considerably improve the accuracy of the estimates (Lemaitre et al. 2012; Simonsen et al. 2011; Thompson et al. 2003, 2009). The Surveillance-Serfling model has the further advantage of fitting data from all seasons and not exclusively from the summer seasons; it thus includes more observation points (i.e., throughout the year), which improves estimates for the single-year age data used in this study. We fitted this model for the P&I underlying causes of mortality in the United States from 1997 to 2016—that is, for the periods during which measures of influenza circulation and mortality are both available on a monthly basis. We tested several models accounting for ILI incidence (CDC 2018), its combination with subtype circulation (ILI decomposed by subtype according to the proportion of positive tests for H1N1, H2N2, and so on), and including a one-month lag term tracking influenza ILI incidence and subtype circulation the month preceding the index month. We selected the best fit for each age based on AIC (Burnham and Anderson 2002). A detailed description of the full model equation, the fitting procedure, and the chosen model parameterization by age are provided in the online appendix.

**Fig. 2:**
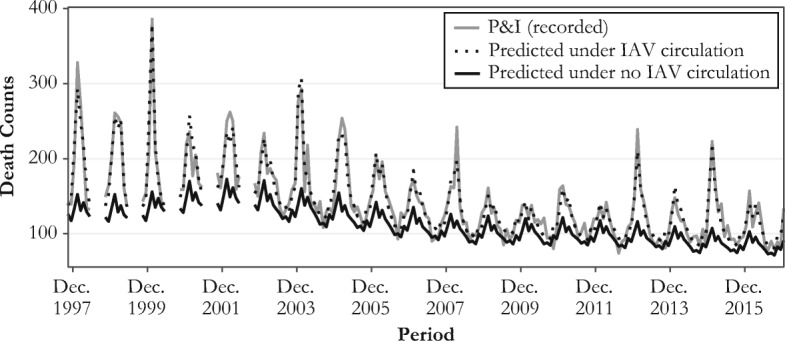
Observed and predicted influenza death counts at age 80, 1997–2016. Between 1998 and 2002, estimates for May–September are not included because there are no influenza circulation data for these periods.

To capture influenza mortality, we first fitted the model to deaths recorded in the P&I categories to obtain a predicted mortality count. Then we set the influenza activity terms (i.e., *flu*_*g*,*t*_ and *flu*_*g*,*t* − 1_ in Eq. (S2)) to 0 to obtain a mortality baseline without influenza activity. The difference between predicted mortality and the baseline reflects mortality caused by influenza. For example, Fig. 2 shows the number of deaths recorded within the P&I category at age 80 between October 1997 and December 2016 (gray line), the number of deaths predicted by the Surveillance-Serfling model given the influenza incidence (dotted line), and the mortality baseline with the influenza terms set to 0 (black line). The distance between the black and the dotted lines is defined as influenza-related mortality.

### Lexis Surfaces

We estimated annual mortality rates over 101 single years of age (ages 0 to 100) and 57 epidemic years (from 1959–1960 through 2015–2016), comprising roughly 5,700 data points. The construction of Lexis surfaces (Vaupel et al. 1998) is done by binding together mortality rates estimated by the Serfling models. To identify differences in mortality levels, a color is assigned to each data point, with the lightest color representing the minimum mortality rate and the darkest representing the maximum. Yearly mortality data were aggregated in our Lexis surfaces by epidemic seasons rather than calendar years—that is, from October 1 to May 31.

### Age-Period-Cohort Analyses

Besides quantifying the influences of each temporal component, statistical APC analyses avoid the subjectivity that may come from visual inspection of Lexis surfaces. Given perfect linear dependence (age = period – cohort), it is impossible, however, to estimate a unique solution describing long-term APC trends without the imposition of additional constraints. Acknowledging this well-known identification problem, we propose two complementary approaches that provide tentative yet heuristic insights on period and cohort trends over sizable stretches of historical time.

More precisely, we first evaluated period and cohort effects according to two opposite scenarios: one in which all the linear trend in mortality change is attributed to period influences (i.e., the cohort effects slope is constrained to 0), and another in which this trend is solely attributed to cohort influences (i.e., the period effects slope is constrained to 0), respectively denoted as the APCd and ACPd scenarios.

Second, we used the intrinsic estimator (IE) method, which finds a solution of the partition of the linear trend between age, period, and cohort by using a constraint that minimizes the APC variance parameter (Fu 2016; Land et al. 2016; Xu and Powers 2016). Given that the constraint is not explicitly chosen by the user, it may be seen as less subjective than other constraints (Yang et al. 2004). Yet, the estimates may vary widely according to the constraints, regardless of whether it is chosen by the user, making the choice of any method ultimately arbitrary.

If there is no unique, statistically optimal solution to partitioning the long-term linear trends in APC models, changes in the slope of these trends are, on the other hand, unambiguously identifiable. These changes provide important information about increases or decreases in mortality risks. For this analysis, we use the *contrast* approach (Tarone and Chu 1996) to identify the breakpoints or “rupture points” where the trend of the cohort effects significantly changes in direction and to quantify these changes (contrasts). For this, we measured the difference between the slopes of two disjoint blocks composed of several consecutive cohorts and assessed their statistical significance according to two alternative approaches. First, we quantified the difference between the slopes formed by the first and last cohorts of each block of cohorts. Alternatively, we compared the sum of all slopes formed by any pair of cohorts contained within each block.

Finally, to reduce the influence of stochastic variation on the APC model estimates, we aggregated data on a two-year basis. To avoid undue influences of seasonal infant and young child mortality that could be unrelated to influenza, such as from the respiratory syncytial virus (Simonsen et al. 2011), we also excluded ages 0–4 from the APC models. See the online appendix for a broader discussion about the use of APC methods, details of the models, and sensitivity analyses. The scripts for the Serfling estimates and the APC analyses are available at https://osf.io/dv9pg/.

## Results

### Influenza Mortality

**Fig. 3:**
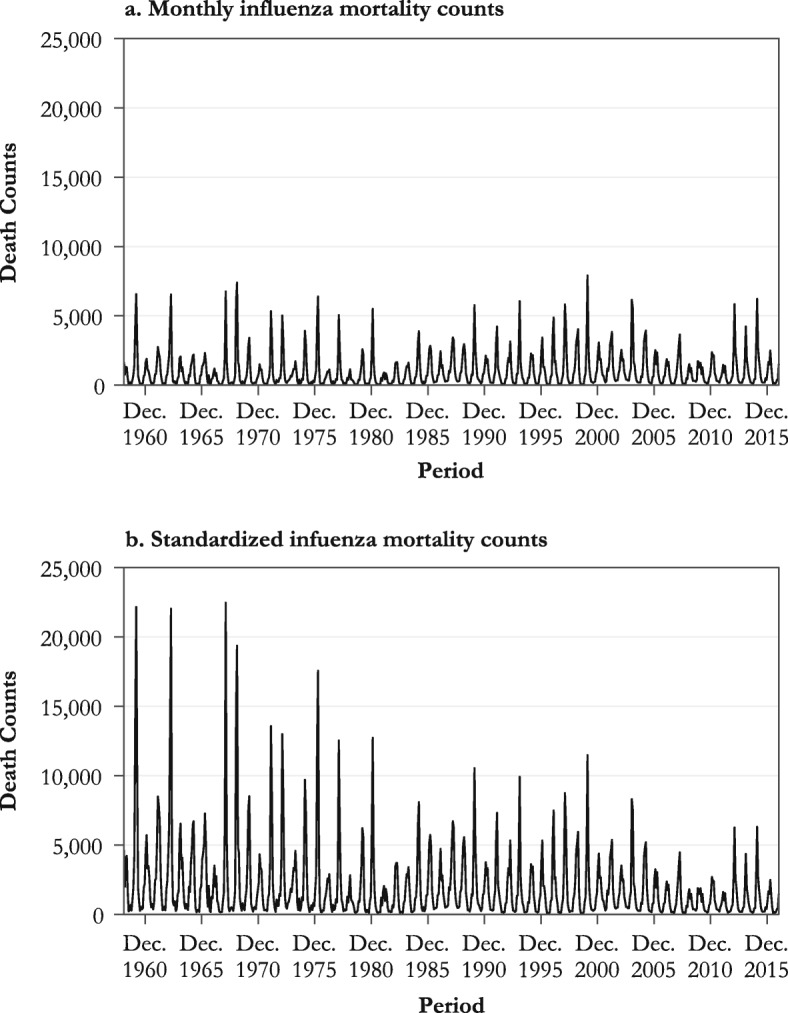
Serfling estimates of monthly influenza death counts (panel a) and of influenza death counts using the total U.S. population in 2015 as the standard population (panel b)

We describe the dynamics of influenza mortality over time by first plotting the monthly influenza mortality counts estimated from the Serfling model over all available calendar months (Fig. 3, panel a). In agreement with previous research, these estimates show substantial mortality variation by period that is related to the dominant virus subtype prevailing during each epidemic season (Reichert et al. 2004; Simonsen et al. 1997; Thompson et al. 2010). For instance, there are noticeable peaks in mortality for the epidemic seasons 1967–1968 and 1999–2000, dominated by the H2N2 and H3N2 influenza subtypes, respectively. By contrast, important mortality dips are apparent for the seasons 1976–1977, 1978–1979, and 2009–2010, respectively dominated by the B, H1N1, and pH1N1 strains. Yet, Fig. 3a shows no clear overall mortality trend over time.

Second, we plot in Fig. 3, panel b, the standardized influenza mortality counts using the July 2015 U.S. total population size and age structure as the standard population (so that results for each year are adjusted to 2015). As shown in the figure, once changes in the size and the age structure of the population are neutralized, a downward trend of influenza mortality appears. For instance, had the 1967 U.S. population shared the size and age structure of the 2015 U.S. population, the number of deaths due to influenza during the whole epidemic season would have been more than three times higher (39,973 vs. 12,463), primarily because older age groups experiencing the highest risk of influenza mortality would have accounted for a larger share of the population.

**Fig. 4:**
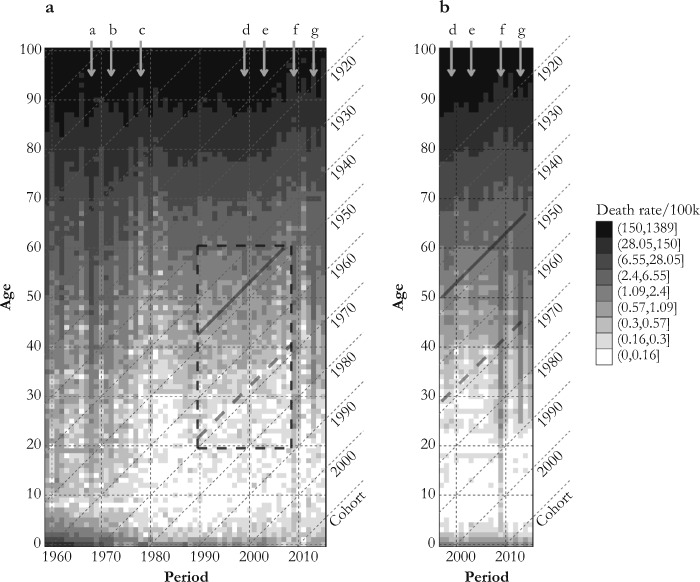
Lexis surfaces of influenza mortality rates estimated by the Serfling model, 1959–2016 (panel a) and the Surveillance-Serfling model, 1997–2016 (panel b). The vertical arrows a, b, d, and e indicate periods of severe H3N2 epidemics. Arrow c marks the reappearance of H1N1 (1977–1978); arrows f and g indicate periods dominated by pH1N1. The solid and dashed black diagonal lines mark the 1947 and 1968 birth cohorts, respectively. The surface covered by the dashed square in panel a is shown in a three-dimensional perspective in Fig. 5.

Table 1 indicates that influenza seasons dominated by the H2N2 subtype (which circulated in the earliest periods covered in this study and disappeared in 1968) were the deadliest, at least based on the U.S. population of 2015 as the standard. Compared with seasons in which the seasonal H1N1 was the dominant subtype, mortality was 2.2 times higher during seasons dominated by the H2N2 subtype, 1.5 times higher for those dominated by H3N2, and 38 % lower in the seasons when the pH1N1 subtype, introduced during the 2009 pandemic, was dominant. If overall mortality was lower during that pandemic (Lemaitre et al. 2012; Nguyen and Noymer 2013; Simonsen et al. 2011), it is mostly due to an overall shift in increased susceptibility from older to younger ages. Young and middle-aged adults aged up to ages 50–60 indeed had increased risks of death during the 2009 outbreak relative to usual influenza seasons, whereas the opposite was true for the elderly (Gagnon et al. 2018a). Contrary to what may be observed during pandemics, as in 2009, influenza mortality was, as expected, higher during normal influenza seasons for the very young or the very old (see upcoming Fig. 4).

**Table 1:** Influenza-associated mortality by dominant viral strain using the size and the age distribution of the total U.S. population in 2015 as the standard population, influenza seasons 1959–2016

Dominant Strain^a^	Average Number of Deaths per Epidemic Season^c^	Standardized Average Number of Deaths per Epidemic Season^d^	Relative Risk of Deaths (ref. = H1N1)^d^
B	8,661	17,504	1.10
H1N1	9,075	15,967	Ref.
pH1N1	9,274	9,946	0.62
H2N2	11,311	34,947	2.19
H3N2	13,024	23,844	1.49
No Dominant Strain^b^	11,056	15,836	0.99

^a^ The dominant strain for a specific season was defined as the strain that cumulated at least 50 % of all isolates identified during that season. H2N2 was imputed as the dominant subtype between 1959 and 1975, when no data about subtype tests are available.

^b^ Influenza seasons for which no subtype reached 50 % of all isolates. Four seasons show no dominant subtype: 1988–1989, 2002–2003, 2006–2007, and 2010–2011.

^c^ Influenza mortality was estimated from the Serfling model applied to P&I mortality data.

^d^ Influenza mortality standardized using the total U.S. population in 2015.

### Lexis Surfaces

Figure 4, panels a and b show Lexis surfaces of influenza mortality estimated with the Serfling and Surveillance-Serfling models. The estimated mortality levels and patterns from both models are highly consistent. Along the age dimension, the surfaces uncover high mortality for the newborn, very low mortality for children and young adults, and a considerable increase in the risk of death after age 50 or 60, as one would expect for this disease.

The surfaces also show high variations of mortality by period, with important mortality surges at all ages during the 1968–1969 pandemic, as well as during the flu seasons of 1972–1973 (Chin et al. 1974), 1999–2000 (CDC 2000), and 2003–2004 (Meadows 2004), all of which resulted from significant drift events of the H3N2 strain (see periods identified by arrows a, b, d, and e at the top of both panels in Fig. 4). Although the 2009 pandemic and the 2013–2014 season were not particularly lethal, they show a mortality shift from older to younger ages (see periods identified with arrows f and g), especially in 2009. As discussed earlier, mortality levels are highly dependent on the predominant virus subtype circulating during each epidemic season. In this sense, the 1960s and 1970s, the second half of the 1990s, as well as the first half of the 2000s are considered as extended periods with high influenza mortality, coinciding with the circulation of the H2N2 and H3N2 subtypes. Conversely, lower mortality is observed after the reappearance of the H1N1 at the end of the 1970s (see period identified with arrow c), especially during the first halves of the 1980s and 1990s, and during the second half of the 2000s, which were dominated by this milder seasonal subtype.

**Fig. 5:**
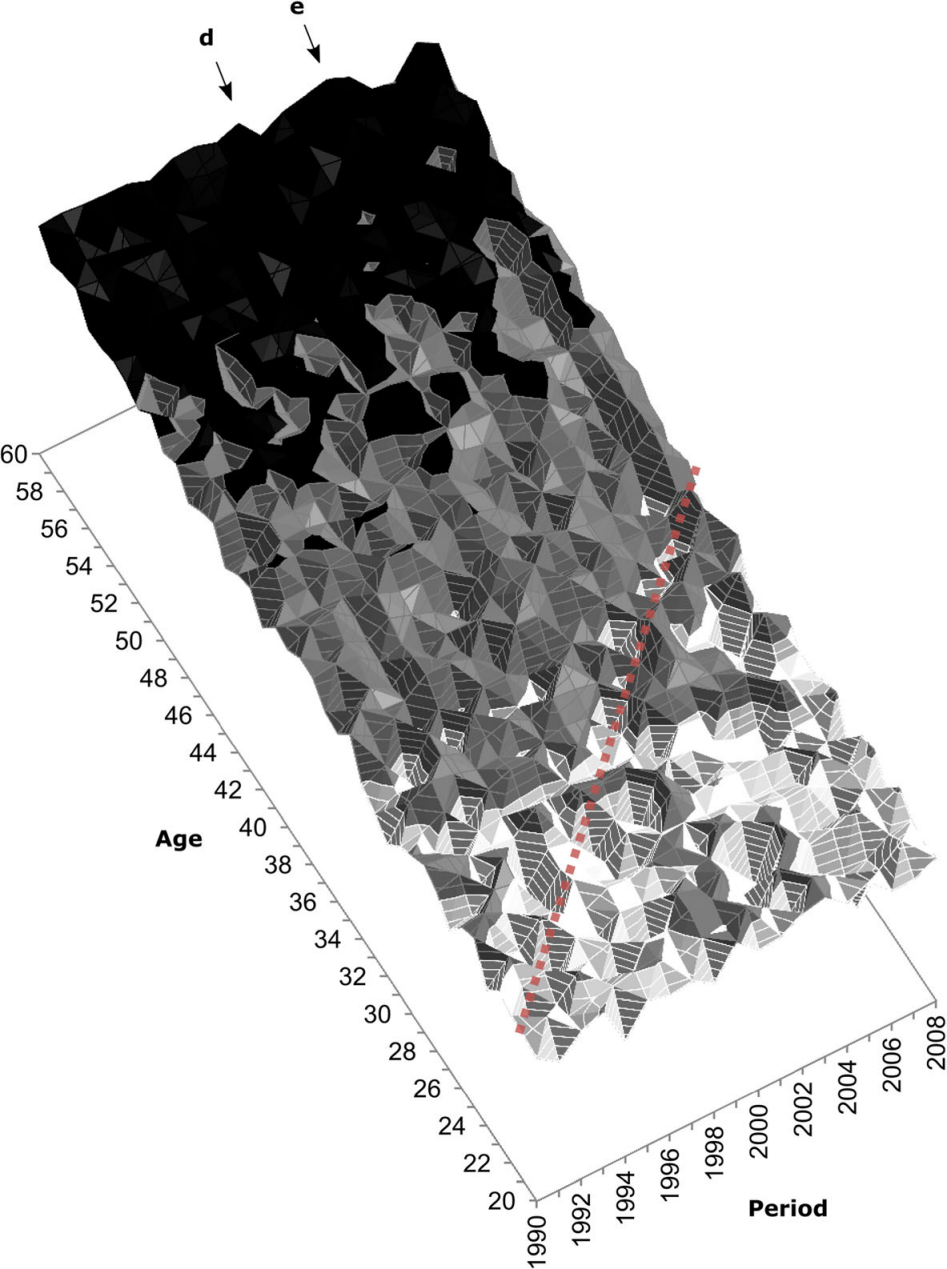
Three-dimensional perspective of the influenza mortality estimated by the Serfling model applied to P&I mortality data. This section frames ages 20–60 and period 1990–2008, covered by the dashed square in Fig. 4, panel a. The dashed diagonal line locates the 1968 birth cohort. Arrows d and e mark severe H3N2 epidemics (see also Fig. 4).

Diagonal patterns in Fig. 4 also suggest the presence of cohort effects. These are immediately perceptible from the 1960s to the beginning of the 1980s, and again from the late 1990s to the late 2000s. Some diagonal patterns are apparent during the milder H1N1 era that spans between those two periods but only at ages below 60. Of note are the cohorts born around the 1968 pandemic (dashed diagonal lines in Figs. 4 and 5), which were presumably exposed early in life to the 1968 H3N2 influenza pandemic virus and which thereafter experienced lower mortality relative to neighboring cohorts (see the light tone “valley” between 1996 and 2006, marked by a dashed diagonal line in Figs. 4 and 5). Figure 4 also suggests the presence of a slight drop in mortality for the 1947 cohort (the tone is generally lighter for this cohort relative to its neighbors, as is clearly the case between 1993 and 1997) and of other “punctual” cohort effects, but further analyses are needed in order to provide firmer support for these observations. The results of our APC analyses presented next help provide such support.

### Linear APC Trends

**Fig. 6:**
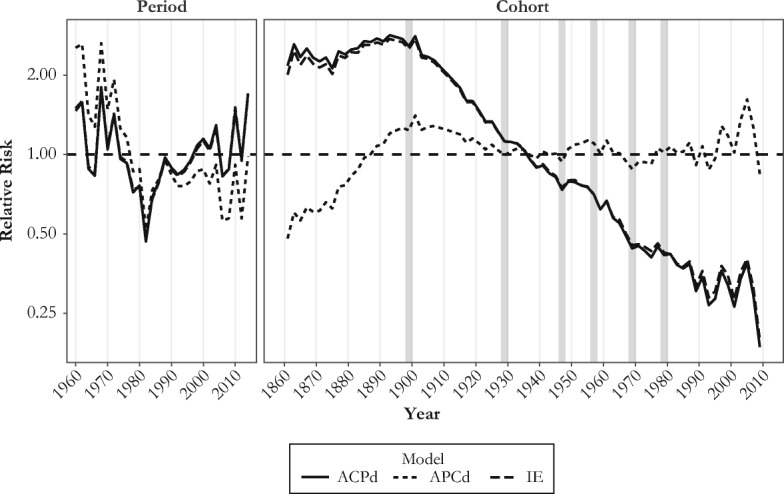
Period and cohort relative (to average) risks of influenza-related mortality derived from the Serfling model, ages 5–100, 1959–2016. The bold gray vertical lines highlight birth cohorts where statistically significant changes in slope occur: that is, 1896–1901, 1928–1929, 1947–1948, 1956–1957, 1968–1969, and 1977–1980 (see Table 2).

Because age-specific effects are regular over time, we focus on period-specific and cohort-specific influences on mortality change. According to our APC detrended model, the long-term slope of mortality change is –0.02024 (*p* < .001). In other words, adjusting for age effects, influenza mortality risk significantly decreased by an average of 2.02 % per year between 1959 and 2016. Figure 6 and Table S5 (online appendix) present APC estimates of influenza mortality derived from two scenarios in which this linear trend is completely attributed either to period influences (APCd, dotted line) or to cohort influences (ACPd, solid line), as well as from the IE model (dashed line).

As expected, the period effect estimates (Fig. 6, left panel; Table S5, upper part) reveal important fluctuations of mortality that closely follow the major antigenic drifts and shifts that took place in the last few decades. Independent of the model’s parametrization, important peaks are immediately visible for the 1968 H3N2 pandemic as well as for the 2003–2004 and 2014–2015 severe H3N2 epidemic seasons. Appreciable dips are also apparent for the 1981–1982, 1993–1994, and 2005–2006 epidemic seasons, during which the dominant subtype was the less virulent H1N1 seasonal virus.

Regarding the long-term linear cohort effects (Fig. 6, right panel; Table S5, lower part), the IE estimates are very similar to those from the ACPd model, suggesting that broadly defined cohort influences mainly account for improvements over time in influenza mortality. The ACPd and the IE estimates both depict a slight increase of mortality throughout cohorts born from 1860 to the late 1890s, followed by a sharp decline from one cohort to the next, continuing to the last decades of the twentieth century. This trend differs markedly from the flatter trend drawn from the APCd estimates, which instead suggests a sizable increase in mortality across cohorts born in the second half of the nineteenth century, followed by a monotonic reduction for cohorts born from around 1900 to the 1930s, and a leveling off thereafter. Note that a method that attributes all the linear trends of mortality to period changes, such as the APCd method, naturally yields a cohort trend that neither increases nor decreases over the period of observation.

Despite substantial differences in the aforementioned scenarios and uncertainties regarding the true trends, some attributes of the cohort effects are common to the three sets of APC estimates: all suggest a decline in the cohort mortality trend for individuals born between 1900 and 1930 and between 1957 and 1968, as well as an increasing trend for years of birth ranging from 1947 to the mid-1950s, and from 1968 to the end of the 1970s. Afterward, the cohort trend might have been either decreasing or leveling off.

### Changes in Trends

Based on visual inspection of Fig. 6, we first identified several ostensible turning points in the cohort effects trend and then investigated these further using the linear contrasts approach. Table 2 lists the six turning points where we identified that changes in the direction of the cohort trend were statistically significant, along with the magnitude of these changes; the turning points are also marked by bold gray vertical lines in the right panel of Fig. 6. Two disjoint blocks composed of 8 to 16 single-year cohorts (i.e., 4 to 8 two-year cohorts) were defined for each breakpoint. We performed the two alternative contrast approaches, denoted *a* and *b*. For five breakpoints out of six, the changes in slope were significant (*p* < .01 or *p* < .05), regardless of the estimated contrast (for one breakpoint, *p* < .10). For simplicity, we focus here on contrast *a*.

**Table 2:** Contrasts for comparing the linear trends between two disjoint blocks of 2-year birth cohorts

Number	Cohorts Where Changes in Slope Occur	Block 1	Block 2	Contrast a	SE	Contrast b	SE
1	~1896–1901	1882–1897	1900–1915	–0.528***	0.085	–5.584***	0.774
2	~1928–1929	1914–1929	1928–1943	0.214*	0.093	1.802*	0.774
3	~1946–1947	1940–1947	1946–1953	0.246**	0.095	0.772**	0.295
4	~1956–1957	1946–1957	1956–1967	–0.430***	0.100	–0.982***	0.221
5	~1968–1969	1960–1969	1968–1977	0.393*	0.156	0.839*	0.337
6	~1976–1981	1968–1977	1980–1989	–0.335*	0.155	–0.585^†^	0.354

*Notes: Contrast a* is defined as the difference between the slopes formed by the straight lines connecting the first and the last pair of consecutive birth cohorts within each block. *Contrast b* is defined as the sum of differences of all slopes formed by any pair of cohorts taken in each block.

^†^*p* < .10; *p < .05; **p < .01; ***p < .001

The first contrast in Table 2 indicates a change in slopes of magnitude –0.528 (*p* < .001) between two blocks composed of 8 two-year cohorts each, indicating a difference of –0.033 in the slope by single-year cohort. This contrast mortality flanks the cohorts born at the turn of the twentieth century (~1896–1901), with the first block including cohorts born from 1882–1883 to 1896–1897, and the second block including those born from 1900–1901 to 1914–1915. The negative contrast indicates a reduction in the slope of the trend of the second block relative to the slope of the first block (see how the curve depicting the cohort effects is concave down in Fig. 6 for the cohorts born at the turn of the twentieth century, regardless of the APC method used). It is worth noting that the crisp peak in Fig. 6 for the cohort 1900–1901 (i.e., a punctual peak flanked by a trough at each side) is the result of systematic misreporting of age (and year of birth) in the death certificates of people born around 1900. Such age heaping, also visible in the form of a diagonal trace in Fig. 4 for the 1900 cohort, is described for influenza mortality during the 1918 influenza pandemic in Ontario in Hallman (2015).

The second contrast indicates an increase of 0.214 (0.013 per year, *p* < .05) in the slope of the cohort trend around 1926–1927. A third contrast indicates a significant upward change of 0.246 (0.031 per year, *p* < .01) in the slope of the trend after the 1946–1947 cohort. The fourth contrast suggests a 0.430 downward change in slope (0.036 per year, *p* < .001) after the 1956–1957 birth cohorts. A fifth contrast reveals a significant 0.393 increase in slope (0.039 per year, *p* < .05) after 1968–1969, and a sixth identifies a decrease of 0.335 (0.034 per year, *p <* .05) in the slope after 1976–1981. The changes in slopes for more recent cohorts were not statistically significant (the small numbers of death make the estimates uncertain). To test the sensitivity of the contrast estimates, we reran the model using three-year instead of two-year cohorts. The results were highly consistent with those presented in Table 2 (see Table S3 in the online appendix).

## Discussion

This study identifies several factors modulating influenza mortality in the U.S. population between 1959 and 2016. Consistent with previous analyses (Reichert et al. 2004; Simonsen et al. 1997; Thompson et al. 2010), the particular IAV subtype circulating during a given season is an important determinant of all-age mortality during that season, with H2N2 being the most lethal subtype, followed by H3N2, H1N1, and pH1N1 (Fig. 3 and Table 1). Over time, the succession and alternation in virus subtypes from one season to the next leaves clear one-year vertical bars on the Lexis configuration that are genuine period effects affecting all age groups simultaneously (vertical arrows in Figs. 4 and 5).

Yet, year-to-year changes in virulence and virus subtype also prime successive cohorts to alternative strains which, through lingering effects on later-life mortality, may leave specific diagonal traces on the Lexis configuration typical of a cohort effect. Our analyses suggest up to three, perhaps four, such “imprinted cohort” effects, centered on the 1947, 1957, 1968, and ~1978–1980 cohorts. The other two significant contrasts identified for the ~1896–1901 and ~1928 cohorts could also be interpreted as imprinted cohort effects, but we believe that they point to longer-term changes in mortality at older ages, in line with the cohort morbidity phenotype. We discuss first the imprinted cohort effects.

The most obvious one concerns the cohorts born at the time of the 1968–1969 “Hong Kong flu” pandemic. Mortality in these cohorts was lower relative to neighboring cohorts, as observed in Figs. 4, 5, and 6, and confirmed by a statistically significant change in slope documented in Table 2. We propose that individuals from the 1968–1969 birth cohorts developed a robust immune response to the H3N2 pandemic virus circulating around the time of their birth. They then benefited from having been primed to that variant when exposed again to subsequent (and numerous) epidemics dominated by the same H3N2 subtype, which has proved to be more lethal than the co-circulating H1N1 variant. However, APC analysis conducted on all-cause mortality and other causes of death (cardiovascular and respiratory) also produced a dip in mortality for cohorts born at the end of the baby boom (Table S4, online appendix). This suggests that the 1968 cohort could also have benefitted from a reduction in influenza mortality unrelated to its early-life antigenic imprinting. Although we cannot fully discard this possibility, we note that the significant contrast identified for influenza mortality is precisely centered on the 1968 cohort in Table S4, whereas the signal for all-cause, cardiovascular, and respiratory mortality is dispersed among cohorts born up to six years before or after 1968.

If the 1968–1969 cohorts benefited from lower mortality relative to neighboring cohorts, the opposite is true for those born around 1978–1980. The increment of influenza risk of death among the cohorts born from 1969 to 1976 likely results from the gradual decrease in the proportion of cohort members primed to the H3N2 subtype. Indeed, with the B subtype, the H1N1—reintroduced into the circulation in early 1978—largely dominated the 1976–1977, 1979–1980, and 1981–1982 flu seasons, and the relative gain in protection that the H1N1-primed cohorts born those years might have had during subsequent outbreaks caused by the H1N1 subtype was more than offset by lack of protection during the more deadly H3N2 outbreaks. Accordingly, the risk could have decreased for cohorts born after 1980 because a higher proportion of individuals among these cohorts were primed to the co-circulating H3N2.

The picture is less clear for the cohorts born around 1957, who were primed to the H2N2 virus that appeared that year, during the so-called Asian flu pandemic. The drop in mortality following that cohort in Fig. 6 and the significant contrasts shown in Table 2 suggest that these cohorts benefitted from cross-protective immunity during the H3N2 seasons through sharing the neuraminidase component N2. Alternatively, the immune systems of the members of this cohorts might have been “refocused” on the H3N2 subtype upon early-life exposure in 1968, adjusting its antigenic signature to this subtype in time, at a relatively young age (Gagnon et al. 2015). Again, the baby boom could have had a role in this context: the 1957 cohort was among the largest of the era, with potential for increased mortality, as described in Easterlin (1987). However, although we find significant contrasts for all-cause and respiratory disease mortality for the 1957 cohort, this signal appeared much more dispersed around that cohort than for influenza mortality (Table S4).

A decade before the Asian flu pandemic, during the 1946–1947 season, a vaccine that was previously effective against the circulating H1N1 virus during the prior seasons totally failed to provide protection because of what turned out to be an important intrasubtypic antigenic drift, akin to a pseudo-pandemic (Kilbourne 2006). Yet, there is no simple immunologic explanation as to why the curve is concave up in Fig. 6 for the cohorts born at that time, quite the contrary given that a deep imprint from H1N1 should instead have increased the risks of mortality in subsequent decades from the H3N2 subtype, as explained above for the 1978–1980 cohorts. Perhaps priming to H1N1 in 1947 was still protective relative to priming to H2N2 in 1957, which was associated with an increase in mortality during the 2009 H1N1 pandemic and the 2013–2014 resurgent outbreak (Gagnon et al. 2018a). However, other mechanisms unrelated to imprinting might have been at play, such as selection. The year 1947 is notorious not only for a major antigenic drift. Demographers have also noted a record-breaking number of marriage licenses issued in May and June 1946 (Whelpton et al. 1947), the first spring season to follow the conclusion of WWII, and therefore a sudden surge of fertility in 1947. Given that the healthiest couples had their first babies within a year following their marriage, the 1947 cohort could have been graced with greater than usual health via synchronization of the fertility of the healthiest parents. Such a “selection-by-synchronization” phenomenon was proposed earlier to explain a surge in the frequency of twin births at the end of WWI in France (Pison et al. 2004).

Of note is the lack of a cohort effect for those born during the 1918 Spanish flu pandemic, which left no specific diagonal trace in the Lexis configuration and produced no significant second-order cohort effects in the contrast analysis. It is possible that selection processes for this cohort offset the lingering effects of early-life exposure to the H1N1 virus that caused the 1918 pandemic. However, its antigenic signature was apparently not fully erased. During the 1968 influenza pandemic, death rate ratios (relative to previous influenza seasons) peaked for the cohorts born around 1918 (Gagnon et al. 2015), while these same cohorts appeared protected during the 2009 flu pandemic (Gagnon et al. 2018a; Jacobs et al. 2012; Nguyen and Noymer 2013).

We hypothesize that the extent to which the lingering effects from early-life imprinting is recognizable depends on the life stage at which mortality is observed. In this study, we observe the cohorts primed to the 1918 virus mostly during seasonal outbreaks, at advanced ages, when influenza deaths would usually no longer directly result from the virus itself, but rather from comorbid conditions and poor health (Plans-Rubió 2007; Reichert et al. 2004; Simonsen et al. 2005), and thus, under the regime of the cohort morbidity phenotype. Accordingly, these differences by age are reflected in the parameterization of the surveillance model in this study. As shown in Table S1 in the online appendix, for ages above 65, the model using a measure of ILI *with no subtype distinction* provided the best fit, whereas for ages 25 to 65, the inclusion of the information related to the virus subtype circulation significantly improved model fit.

This brings us to the contrasts identified for the cohorts born around 1900 and 1928 (see Table 2). The two years are often considered as years of significant antigenic drifts (Beveridge 1991; Collins 1931; Patterson 1986). Although the status of the first is debated (Hill et al. 2017), there is potential for long-term imprinted cohort effects for individuals born during those two years, as described in this study for more recent cohorts. However, we believe that the contrasts for these cohorts born in the first half of the twentieth century (observed at older ages in this study) are rather mainly indicative of broader transition processes.

Researchers have extensively documented a context of deprived sanitary conditions in Northern America during the late nineteenth century due to the rapid growth of cities, reflected in high infant and child mortality (Burian et al. 2000; Haines 2000; Olson and Thornton 2011). Despite important discoveries in bacteriology in the nineteenth century, germ theory did not begin to guide public policies until the turn of the twentieth century. As Preston and Haines (1991:22) argued, “There is probably no area of public health where a majority of the progress between 1850 and 1950 occurred by 1900.” However, between 1900 and 1930, the United States experienced the most rapid decline in mortality rates in documented history as a result of sharp reductions in infant mortality from infectious diseases (Cutler and Miller 2005). This is consistent with the downward trend in cohort effects between these dates: progressively lower levels of disease load early in life translated into lower and lower levels of influenza mortality later in life.

Congruently, a distinctive second-order linear contrast around 1900 clearly marks the beginning of this trend (Table 2). This contrast remained highly significant when moving the rupture point forward or backward by up to six years (Table S4 shows constant contrasts up to four years forward or backward), suggesting that it is not associated to one specific antigenic drift year but to a smoother and longer-term change. Because the contrast identified about 30 years later is not uniquely centered on the 1928 cohort, it could signal the end of the sharp decline in influenza mortality that began with the cohorts born at the turn of the twentieth century. Had this contrast mainly resulted from the 1928 antigenic event, the changes in the slope would also have appeared more abrupt, more statistically significant, and concentrated on a single year, as is the case for the subsequent contrasts. Admittedly, it is not possible with the data at hand to provide firmer evidence regarding the nature of the contrasts identified. It is also possible that both antigenic imprinting and cohort morbidity phenotype scenarios are at play in the earlier cohorts of this study.

Our study has several limitations. We discuss three. First, Surveillance-Serfling models did not include information about other viruses that could be correlated with influenza seasonality, such as respiratory syncytial virus. Consequently, our estimations of influenza mortality could be slightly overestimated. Nonetheless, this potential bias would be mostly concentrated in children under age 5 (Simonsen et al. 2011), which were excluded from the APC analysis. In addition, infections with that virus are unlikely to explain mortality trends that coincide with the circulation of specific strains of influenza virus in a given season.

Second, mortality data used here are useful for identifying age-related trends but are limited concerning other concomitant influences affecting disease burden and mortality risks, such as medical comorbidity, propensity for care-seeking, laboratory testing of viral strains, or even infection rates, which may break down differently by population subgroups. Our choice to focus on P&I deaths as a basis to estimate the number of influenza deaths most likely led to an underestimation of this number, especially at older ages (because of comorbidities). This means that the old-young difference is probably biased. Had we used a broader category—such as “cardiorespiratory”—our model would have proportionally captured more deaths from the exacerbation of comorbidities at older than at younger ages. Other important factors could affect mortality outcomes, notably race or sex. Haines noted, for instance, that African Americans were protected to some extent by their more rural residence in the first half of the twentieth century, although their mortality remained higher than that of other Americans (Haines 2000, 2001). It is thus possible that the African Americans who were born during those years and who survived to the onset of this study in 1959 had a shallower imprint to specific influenza strains than the general population because of lower incidence of influenza in early life, with potentially less clear antigenic imprinting effects in these groups. Unfortunately, because of sample size issues, we could not explore such a possibility in the context of this study. Regarding sex differences, men are often said to declare more (and complain more from) flu symptoms than women, which is popularly encapsulated in the expression “man flu,” perhaps erroneously (Sue 2017). Relative to women, they also appear to be less responsive to influenza vaccination and to be in general more susceptible to complications and death to many acute respiratory diseases, including influenza (Engler et al. 2008; Furman et al. 2014; Giefing-Kröll et al. 2015). However, our analyses could not reveal fundamental sex-based differences with respect to APC patterns of influenza mortality (not shown here).

Third, and perhaps most importantly, the use of first-order APC statistical analysis has been criticized as unreliable because of the irresolvable issue of full dependency of the three age, period, and cohort components, which are sensitive to restrictions arbitrarily imposed (Luo 2013; Luo et al. 2016; O’Brien 2013). Yet, although perfect collinearity will never be overcome, we believe it is still possible to gain useful knowledge about cohort trends, especially if several methods are used and if the analyses are supported and informed by external evidence from history and the social sciences in general (Luo 2013). For instance, based on Fig. 6, it is unclear whether mortality increased in cohorts born from 1850 to 1900 or whether it remained relatively stable. But the same figure shows that three models with widely different sets of constraints concur to show declining mortality in cohorts born from 1900 to 1930. This pattern can also be seen in the Lexis surfaces of Figs. 4, and taking into consideration the historical sketch above, we feel it would be difficult to argue that period-based rather than cohort-based factors explain this trend. That said, results for long-term linear trends should always be seen as indicative or exploratory, not as confirmatory. For recent cohorts, it is no surprise that uncertainties persist about cohort versus period long-term trends on mortality; these cohorts were observed for only a short time at relatively young ages, when mortality risks are relatively low.

## Conclusion

The findings reported in this study have several key implications. On the one hand, they suggest that the mechanisms proposed by the antigenic imprinting and the cohort morbidity phenotype hypotheses are not necessarily mutually exclusive as engines of influenza mortality variation; these two mechanisms even seem to act simultaneously, triggering different mortality changes at distinct levels or scales. Yet, the irregular and sudden changes in influenza mortality at young ages are largely caused by the interactions between the population’s signature of antigenic imprinting and the characteristics of the virus encountered in adulthood. The progressive decline in influenza mortality observed in cohorts born between 1900 and 1930 (and for virtually all cohorts born after 1900 if we accept the IE results), on the other hand, would result from continuous improvements of early-life conditions (better hygiene, lower disease load in infancy, and so on), which manifest themselves at older ages.

We suggest that the contrasting mortality patterns reflect a difference in the pathways that lead from influenza infection to death at different ages and that this difference has major health policy implications. Interventions for younger patients should be focused on mitigating the immune response when this response is potentially harmful (e.g., during pandemics). On the other hand, in the case of the elderly, indirect pathways involving comorbid conditions should be targeted as priorities. It is also important that vaccination campaigns cease to identify susceptible groups of individuals based almost exclusively on their age group, and instead define susceptibility from a combination of APC influences. The yearly defined cohort effects and contrasts are further evidence that surveillance and mortality data on influenza should be made available by single years of age to all stakeholders, as Gagnon et al. (2018b) argued. Knowing the strain to which a cohort has been primed and how “deep” this antigenic signature is would help to improve the efficiency of immunization campaigns and to inform medical professionals about priorities based on the age or the generation of patients.

The finding that cohort-specific influences may account for important changes in influenza mortality at older ages also tempers the common assumption that reductions in mortality from infectious diseases stemmed exclusively from period-based improvements in environmental and technological factors such as sanitation, hygienic practices, and medical technology. We argue that a considerable part of improvements, at least for the cohorts born between 1900 and 1930, was accomplished on a cohort basis. In this context, the general increase in overall mortality from influenza, which is expected in the coming years because of population aging (Simonsen et al. 2011), might be tempered by long-term beneficial effects of earlier improvements in early-life conditions. Therefore, our results highlight the importance of these conditions not only for the reduction of chronic and degenerative mortality but also for the enhancement of survival from infectious diseases at old ages. It would be interesting to perform similar analyses on other infectious diseases to assess the generalizability of the scenarios proposed here.

## Electronic supplementary material

ESM 1 (PDF 1723 kb)

## References

[CR1] Almond D (2006). Is the 1918 Influenza pandemic over? Long-term effects of in utero influenza exposure in the post-1940 U.S. population. Journal of Political Economy.

[CR2] Anderson, R. N., Miniño, A. M., Hoyert, D. L., & Rosenberg, H. M. (2001). *Comparability of cause of death between ICD-9 and ICD-10: Preliminary estimates* (National Vital Statistics Reports, Vol. 49 No. 2). Hyattsville, MD: National Center for Health Statistics. 11381674

[CR3] Armstrong GL, Conn LA, Pinner RW (1999). Trends in infectious disease mortality in the United States during the 20th century. JAMA.

[CR4] Azambuja MIR (2009). Influenza recycling and secular trends in mortality and natality. British Actuarial Journal.

[CR5] Azambuja, M. I. (2015, September). *Use of 1-year intervals in graphic plots of age-period-cohort trends suggests a role for influenza in secular (period and cohort) variations of all-causes mortality*. Paper presented at the Workshop of the EAPS: Health, Morbidity and Mortality Working Group, Prague, Czech Republic.

[CR6] Barry JM (2005). The Great Influenza: The epic story of the deadliest plague in history.

[CR7] Beveridge WIB (1991). The chronicle of influenza epidemics. History and Philosophy of the Life Sciences.

[CR8] Burian SJ, Nix SJ, Pitt RE, Durrans SR (2000). Urban wastewater management in the United States: Past, present, and future. Journal of Urban Technology.

[CR9] Burnham KP, Anderson DR (2002). Model selection and multimodel inference: A practical information-theoretic approach.

[CR10] Canudas-Romo V, Guillot M (2015). Truncated cross-sectional average length of life: A measure for comparing the mortality history of cohorts. Population Studies.

[CR11] Carstensen B (2007). Age-period-cohort models for the Lexis diagram. Statistics in Medicine.

[CR12] Centers for Disease Control (CDC). (2000). Update: Influenza activity—United States, 1999–2000 season. Morbidity and Mortality Weekly Report.

[CR13] Centers for Disease Control (CDC). (2018). *FluView: National, regional, and state level outpatient illness and viral surveillance* [Data set]. Available from https://gis.cdc.gov/grasp/fluview/fluportaldashboard.html

[CR14] Chin J, Magoffin RL, Lennette EH (1974). The epidemiology of influenza in California, 1968–1973. Western Journal of Medicine.

[CR15] Clayton D, Schifflers E (1987). Models for temporal variation in cancer rates. II: Age–period–cohort models. Statistics in Medicine.

[CR16] Cobey, S., & Hensley, S. E. (2017). Immune history and influenza virus susceptibility. *Current Opinion in Virology, 22,* 105–111. 10.1016/j.coviro.2016.12.004 PMC5467731 28088686

[CR17] Cohen SA, Klassen AC, Ahmed S, Agree EM, Louis TA, Naumova EN (2010). Trends for influenza and pneumonia hospitalization in the older population: Age, period, and cohort effects. Epidemiology and Infection.

[CR18] Collins SD (1931). Age and sex incidence of influenza and pneumonia morbidity and mortality in the epidemic of 1928–29 with comparative data for the epidemic 1918–19. Public Health Reports.

[CR19] Cutler D, Miller G (2005). The role of public health improvements in health advances: The twentieth-century United States. Demography.

[CR20] Davenport FM, Hennessy AV, Francis T, Fabisch P (1953). Epidemiologic and immunologic significance of age distribution of antibody to antigenic variants of influenza virus. Journal of Experimental Medicine.

[CR21] Deaton A (2015). The great escape: Health, wealth, and the origins of inequality.

[CR22] Easterlin RA (1987). Birth and fortune: The impact of numbers on personal welfare.

[CR23] Engler Renata J. M. (2008). Half- vs Full-Dose Trivalent Inactivated Influenza Vaccine (2004-2005). Archives of Internal Medicine.

[CR24] Finch CE, Crimmins EM (2004). Inflammatory exposure and historical changes in human life-spans. Science.

[CR25] Floud R, Fogel RW, Harris B, Hong SC (2011). The changing body: Health, nutrition, and human development in the western world since 1700.

[CR26] Fogel RW, Costa DL (1997). A theory of technophysio evolution, with some implications for forecasting population, health care costs, and pension costs. Demography.

[CR27] Fu W (2016). Constrained estimators and consistency of a regression model on a Lexis diagram. Journal of the American Statistical Association.

[CR28] Fu Wenjiang J. (2000). Ridge estimator in singulah oesiun with application to age-period-cohort analysis of disease rates. Communications in Statistics - Theory and Methods.

[CR29] Furman D., Hejblum B. P., Simon N., Jojic V., Dekker C. L., Thiebaut R., Tibshirani R. J., Davis M. M. (2013). Systems analysis of sex differences reveals an immunosuppressive role for testosterone in the response to influenza vaccination. Proceedings of the National Academy of Sciences.

[CR30] Gagnon, A., Acosta, E., Hallman, S., Bourbeau, R., Dillon, L. Y., Ouellette, N., . . . Miller, M. S. (2018a). Pandemic paradox: Early life H2N2 pandemic influenza infection enhanced susceptibility to death during the 2009 H1N1 pandemic. *mBio, 9*(1), e02091–17. 10.1128/mBio.02091-17 10.1128/mBio.02091-17 PMC5770550 29339427

[CR31] Gagnon A, Acosta E, Miller MS (2018). Reporting and evaluating influenza virus surveillance data: An argument for incidence by single year of age. Vaccine.

[CR32] Gagnon A, Acosta JE, Madrenas J, Miller MS (2015). Is antigenic sin always “original?” Re-examining the evidence regarding circulation of a human H1 Influenza virus immediately prior to the 1918 Spanish Flu. PLoS Pathogens.

[CR33] Gagnon A, Miller MS, Hallman SA, Bourbeau R, Herring DA, Earn DJ, Madrenas J (2013). Age-specific mortality during the 1918 Influenza Pandemic: Unravelling the mystery of high young adult mortality. PLoS One.

[CR34] Giefing-Kröll C, Berger P, Lepperdinger G, Grubeck-Loebenstein B (2015). How sex and age affect immune responses, susceptibility to infections, and response to vaccination. Aging Cell.

[CR35] Haines M, Engerman SL, Gallman RE (2000). The population of the United States, 1790–1920. The Cambridge economic history of the United States.

[CR36] Haines MR (2001). The urban mortality transition in the United States, 1800–1940. Annales de Demographie Historique.

[CR37] Hallman, S. A. (2015). The demographic links between the 1890 and 1918 Influenza Pandemics in Ontario. *Electronic Thesis and Dissertation Repository, 3129*. Retrieved from http://ir.lib.uwo.ca/etd/3129

[CR38] Hallman, S. A., & Gagnon, A. (2014). Does exposure to influenza very early in life affect mortality risk during a subsequent outbreak? The 1890 and 1918 pandemics in Canada. In M. K. Zuckerman (Ed.), *Modern environments and human health? Revisiting the second epidemiological transition* (pp. 123–138). Hoboken, NJ: Wiley-Blackwell.

[CR39] Haque A, Hober D, Kasper LH (2007). Confronting potential influenza A (H5N1) pandemic with better vaccines. Emerging Infectious Diseases.

[CR40] Henry C, Palm A-KE, Krammer F, Wilson PC (2018). From original antigenic sin to the universal influenza virus vaccine. Trends in Immunology.

[CR41] Hill EM, Tildesley MJ, House T (2017). Evidence for history-dependence of influenza pandemic emergence. Scientific Reports.

[CR42] Holford TR (1991). Understanding the effects of age, period, and cohort on incidence and mortality rates. Annual Review of Public Health.

[CR43] Human Mortality Database. (2019). University of California, Berkeley (USA), and Max Planck Institute for Demographic Research (Germany). Available from http://www.mortality.org/

[CR44] Jacobs Jessica Hartman, Archer Brett Nicholas, Baker Michael G., Cowling Benjamin J., Heffernan Richard T., Mercer Geoff, Uez Osvaldo, Hanshaoworakul Wanna, Viboud Cécile, Schwartz Joel, Tchetgen Tchetgen Eric, Lipsitch Marc (2012). Searching for Sharp Drops in the Incidence of Pandemic A/H1N1 Influenza by Single Year of Age. PLoS ONE.

[CR45] Kelly E (2009). *The scourge of Asian Flu: In utero exposure to pandemic influenza and the development of a cohort of British children* (IFS Working Paper, No. W09/17).

[CR46] Kilbourne ED (2006). Influenza pandemics of the 20th century. Emerging Infectious Diseases.

[CR47] Klebba AJ, Dolman AB (1975). Comparability of mortality statistics for the seventh and eighth revisions of the International Classification of Diseases, United States.

[CR48] Klebba AJ, Scott J (1980). *Estimates of selected comparability ratios based on dual coding of 1976 death certificates by the eighth and ninth revisions of the International Classification of Diseases* (Monthly Vital Statistics Report, No. 28/11).

[CR49] Kobasa Darwyn, Jones Steven M., Shinya Kyoko, Kash John C., Copps John, Ebihara Hideki, Hatta Yasuko, Hyun Kim Jin, Halfmann Peter, Hatta Masato, Feldmann Friederike, Alimonti Judie B., Fernando Lisa, Li Yan, Katze Michael G., Feldmann Heinz, Kawaoka Yoshihiro (2007). Aberrant innate immune response in lethal infection of macaques with the 1918 influenza virus. Nature.

[CR50] Land KC, Fu Q, Guo X, Jeon SY, Reither EN, Zang E (2016). Playing with the rules and making misleading statements: A response to Luo, Hodges, Winship, and Powers. American Journal of Sociology.

[CR51] Lemaitre M, Carrat F, Rey G, Miller M, Simonsen L, Viboud C (2012). Mortality burden of the 2009 A/H1N1 Influenza pandemic in France: Comparison to seasonal influenza and the A/H3N2 Pandemic. PLoS One.

[CR52] Loo Y-M, Gale M (2007). Influenza: Fatal immunity and the 1918 virus. Nature.

[CR53] Luo L (2013). Assessing validity and application scope of the intrinsic estimator approach to the age-period-cohort problem. Demography.

[CR54] Luo L, Hodges J, Winship C, Powers D (2016). The sensitivity of the intrinsic estimator to coding schemes: Comment on Yang, Schulhofer-Wohl, Fu, and Land. American Journal of Sociology.

[CR55] Ma J, Dushoff J, Earn DJD (2011). Age-specific mortality risk from pandemic influenza. Journal of Theoretical Biology.

[CR56] Mazumder B, Almond D, Park K, Crimmins EM, Finch CE (2010). Lingering prenatal effects of the 1918 Influenza pandemic on cardiovascular disease. Journal of Developmental Origins of Health and Disease.

[CR57] Meadows M (2004). A look at the 2003–2004 flu season. FDA Consumer.

[CR58] Meslé F, Vallin J (2000). Transition sanitaire: Tendances et perspectives [Sanitary transition: Trends and perspectives]. Revue Médecine/Sciences.

[CR59] Miller MS, Gardner TJ, Krammer F, Aguado LC, Tortorella D, Basler CF, Palese P (2013). Neutralizing antibodies against previously encountered influenza virus strains increase over time: A longitudinal analysis. Science Translational Medicine.

[CR60] NCHS. (2018). *Vital Statistics online data portal* [Public use data files]. Available from http://www.cdc.gov/nchs/data_access/Vitalstatsonline.htm

[CR61] Nelson MI, Holmes EC (2007). The evolution of epidemic influenza. Nature Reviews Genetics.

[CR62] Nguyen AM, Noymer A (2013). Influenza mortality in the United States, 2009 pandemic: Burden, timing and age distribution. PLoS One.

[CR63] Noymer A, Nguyen AM (2013). Influenza as a proportion of pneumonia mortality: United States, 1959–2009. Biodemography and Social Biology.

[CR64] O’Brien Robert M. (2013). Comment of Liying Luo’s Article, “Assessing Validity and Application Scope of the Intrinsic Estimator Approach to the Age-Period-Cohort Problem”. Demography.

[CR65] Oeppen, J., & Wilson, C. (2006, March–April). *Epidemiological evidence for viral exposure in childhood as a risk-factor in subsequent influenza pandemics*. Paper presented at the annual meeting of the Population Association of America, Los Angeles, CA.

[CR66] Olson S, Thornton P (2011). Peopling the North American city: Montreal, 1840–1900.

[CR67] Patterson KD (1986). Pandemic influenza, 1700–1900: A study in historical epidemiology.

[CR68] Pison G, Couvert N, Wasserman MR (2004). The frequency of twin births in France. Population.

[CR69] Plans-Rubió P (2007). Prevention and control of influenza in persons with chronic obstructive pulmonary disease. International Journal of Chronic Obstructive Pulmonary Disease.

[CR70] Preston SH, Haines MR (1991). Fatal years: Child mortality in late nineteenth-century America.

[CR71] Rajendran, M., Nachbagauer, R., Ermler, M. E., Bunduc, P., Amanat, F., Izikson, R., . . . Krammer, F. (2017). Analysis of anti-influenza virus neuraminidase antibodies in children, adults, and the elderly by ELISA and enzyme inhibition: Evidence for original antigenic sin. *mBio, 8*(2), e02281–16. 10.1128/mBio.02281-16 10.1128/mBio.02281-16 PMC5362038 28325769

[CR72] Rau, R., Bohk, C., Muszynska, M. M., & Vaupel, J. W. (2013, April). *Rates of mortality improvement on the Lexis surface: Visualizing age-, period-, and cohort-effects*. Paper presented at the annual meeting of the Population Association of America, New Orleans, LA.

[CR73] Reichert TA, Simonsen L, Sharma A, Pardo SA, Fedson DS, Miller MA (2004). Influenza and the winter increase in mortality in the United States, 1959–1999. American Journal of Epidemiology.

[CR74] Serfling RE (1963). Methods for current statistical analysis of excess pneumonia-influenza deaths. Public Health Reports.

[CR75] Shanks GD, Brundage JF (2012). Pathogenic responses among young adults during the 1918 Influenza pandemic. Emerging Infectious Diseases.

[CR76] Simonsen L, Clarke MJ, Schonberger LB, Arden NH, Cox NJ, Fukuda K (1998). Pandemic versus epidemic influenza mortality: A pattern of changing age distribution. Journal of Infectious Diseases.

[CR77] Simonsen L, Clarke MJ, Williamson GD, Stroup DF, Arden NH, Schonberger LB (1997). The impact of influenza epidemics on mortality: Introducing a severity index. American Journal of Public Health.

[CR78] Simonsen L, Reichert TA, Viboud C, Blackwelder WC, Taylor RJ, Miller MA (2005). Impact of influenza vaccination on seasonal mortality in the US elderly population. Archives of Internal Medicine.

[CR79] Simonsen L, Viboud C, Taylor RJ, Miller MA, Rappuoli R, Giudice GD (2011). The epidemiology of influenza and its control. Influenza vaccines for the future.

[CR80] Smith DJ, Forrest S, Ackley DH, Perelson AS (1999). Variable efficacy of repeated annual influenza vaccination. Proceedings of the National Academy of Sciences.

[CR81] Sue, K. (2017). The science behind “man flu.” *BMJ, 359,* j5560. 10.1136/bmj.j5560 10.1136/bmj.j5560 29229663

[CR82] Tarone R, Chu KC (1996). Evaluation of birth cohort patterns in population disease rates. American Journal of Epidemiology.

[CR83] Taubenberger JK, Morens DM (2006). 1918 Influenza: The mother of all pandemics. Emerging Infectious Diseases.

[CR84] Thompson, M. G., Shay, D. K., Zhou, H., Bridges, C. B., Cheng, P. Y., Burns, E., . . . CDC. (2010). Estimates of deaths associated with seasonal influenza—United States, 1976–2007. *Morbidity and Mortality Weekly Report, 59,* 1058–1062. 20798667

[CR85] Thompson WW, Shay DK, Weintraub E, Brammer L, Cox N, Anderson LJ, Fukuda K (2003). Mortality associated with influenza and respiratory syncytial virus in the United States. JAMA.

[CR86] Thompson William W., Weintraub Eric, Dhankhar Praveen, Cheng Po-Yung, Brammer Lynnette, Meltzer Martin I., Bresee Joseph S., Shay David K. (2009). Estimates of US influenza-associated deaths made using four different methods. Influenza and Other Respiratory Viruses.

[CR87] Vaupel JW, Zhenglian W, Andreev KF, Yashin AI (1998). Population data at a glance: Shaded contour maps of demographic surfaces over age and time.

[CR88] Viboud Cecile, Miller Mark, Olson Donald R, Osterholm Michael, Simonsen Lone (2010). Preliminary Estimates of Mortality and Years of Life Lost Associated with the 2009 A/H1N1 Pandemic in the US and Comparison with Past Influenza Seasons. PLoS Currents.

[CR89] Whelpton PK, Eldridge HT, Seigel JS, U.S. Bureau of the Census (1947). Forecasts of the population of the United States, 1945–1975.

[CR90] Worobey M, Han G-Z, Rambaut A (2014). Genesis and pathogenesis of the 1918 pandemic H1N1 Influenza A virus. Proceedings of the National Academy of Sciences.

[CR91] Xu M, Powers DA, Schoen R (2016). Bayesian ridge estimation of age-period-cohort models. Dynamic demographic analysis.

[CR92] Yang Y, Fu WJ, Land KC (2004). A methodological comparison of age-period-cohort models: The intrinsic estimator and conventional generalized linear models. Sociological Methodology.

