# Supplementary material for: Determinants of Influenza Mortality Trends: Age-Period-Cohort Analysis of Influenza Mortality in the United States, 1959–2016
Source: Demography. 2019 Sep 9;56(5):1723–46. doi: 10.1007/s13524-019-00809-y (PMC6797638; doi:10.1007/s13524-019-00809-y)
Supplement: Supplementary file 1 — (PDF 1723 kb) [file 13524_2019_809_MOESM1_ESM.pdf]

Determinants of Influenza Mortality Trends:

Age-Period-Cohort Analysis of Influenza Mortality in the United States, 1959-2016

Online Appendix

Influenza Mortality Models

The Serfling Regression Model

We used a Serfling regression model in order to estimate mortality from influenza from 1959 to 2016 and to explore its age, period, and cohort components. We first estimated a mortality baseline without influenza by fitting Pneumonia and Influenza (P&I) death counts during the summer season, during which the influenza virus does not circulate widely in North America. Influenza-related mortality was estimated for each month as the difference between the observed P&I death count and the estimated baseline. Since previous analyses have used different combinations of summer months to define the baseline (Dushoff et al. 2006; Lemaitre et al. 2012; Nguyen and Noymer 2013; Simonsen et al. 2005), we tested four summer periods to fit the Serfling model (i.e., May to September, May to October, June to September, and June to October). The comparison of these estimates with those obtained from the Surveillance-Serfling model, which are estimated over the whole year (see below), guided us in selecting the summer period for the Serfling model (see Fig. S1).

The formulation of our Serfling model is:

$$log(deaths_{a,t}) = \sum_{i=0}^{10} \beta_i t^i + \beta_{11} sin\left(\frac{2\pi t}{12}\right) + \beta_{12} cos\left(\frac{2\pi t}{12}\right) + log(exposure_{a,t}), \qquad \text{(S1)}$$

where a is age (a = 0, 1, 2, …, 100), t the epidemic period (here from January 1959 to December 2016), $deaths_{a,t}$ the death count, and $exposure_{a,t}$ the population at risk. The model thus includes three key components: ($\sum_{i=0}^{10} \beta_i t^i$) controls for secular trends in mortality, while ($\beta_{11} sin\left(\frac{2\pi t}{12}\right) + \beta_{12} cos\left(\frac{2\pi t}{12}\right)$) captures influenza seasonality over time, and $log(exposure_{a,t})$ controls for changes in the age structure of the population over time.

In contrast to the original formulation and common uses of the Serfling method, which is based on linear regression models (Serfling 1963) or Poisson distributions (Thompson et al. 2009), we used a negative binomial distribution to estimate this model, which accounts for overdispersion. This distribution is also better-suited for low-frequency-count data (Hilbe 2011; Nguyen and Noymer 2013), which may indeed occur given the single-year age classification used here.

To estimate the mortality baseline for each age and summer period definition, we tested nine different polynomial degrees for the secular trend (from the 2nd to the 10th) and two seasonal terms (*sin* and *sin* + *cos*). Based on the Akaike information criteria (AIC) we selected the model providing the best fit among the 18 alternative parameterizations. The threshold values proposed by Hilbe (2011) were used to assist in deciding if the improvement of the model fit was statistically significant.

The Surveillance-Serfling Model

In order to estimate influenza mortality, we also use a "Surveillance-Serfling" regression model, which includes parameters tracking influenza-like illness (ILI) incidence and influenza circulation by subtype between 1997 and 2016. The model is written as:

$$log(deaths_{a,t}) = \sum_{i=0}^{10} \beta_i t^i + \beta_{11} sin\left(\frac{2\pi t}{12}\right) + \beta_{12} cos\left(\frac{2\pi t}{12}\right) + \beta_{13} sin\left(\frac{3\pi t}{12}\right) + \beta_{14} cos\left(\frac{3\pi t}{12}\right) + \beta_{15} sin\left(\frac{4\pi t}{12}\right) + \beta_{16} cos\left(\frac{4\pi t}{12}\right) + \beta_{17} sin\left(\frac{6\pi t}{12}\right) + \beta_{18} cos\left(\frac{6\pi t}{12}\right) + \beta_{19} sin\left(\frac{8\pi t}{12}\right) + \beta_{20} cos\left(\frac{8\pi t}{12}\right) + \beta_{21} sin\left(\frac{10\pi t}{12}\right) + \beta_{22} cos\left(\frac{10\pi t}{12}\right) + \beta_{23} flu_{g,t} + \beta_{24} flu_{g,t-1} + log(exposure_{a,t}), \quad (S2)$$

where a is age (a = 0, 1, 2, …, 100), t the monthly period (over 211 months, from October 1997 to December 2016, excluding periods from May through September between 1998 and 2002, for which influenza circulation data is not available), $deaths_{a,t}$ the death counts, and $exposure_{a,t}$ the population at risk. Like the traditional Serfling model, this model controls for secular trends in mortality ($\sum_{i=0}^{10} \beta_i t^i$) and seasonality (with the *sin/cos* terms), while $log(exposure_{a,t})$ tracks changes in the age structure of the population over time.

In addition, $flu_{i,g,t}$ and $flu_{i,g,t-1}$ account for influenza virus circulation during the current (*t*) and the previous (*t-1*) month, respectively. To define the measure of virus circulation, we tested several options, with models that included influenza-like illness (ILI) incidence terms by age group *g* (*g* = 0, 1-4, 5-24, 25-64, 65+), combined with influenza surveillance data by subtype.

The measure accounting for ILI incidence by age group is defined for the current month *t* as:

$$flu_{g,t} = ILI_t * \frac{op_{g,t}}{Top_t}. \quad (S3)$$

For each month *t*, ILI_t is the percentage of outpatients with ILI symptoms, $op_{g,t}$ the numbers of outpatients of age group g with ILI symptoms, and Top_t the numbers of outpatients of all ages with ILI symptoms.

Alternatively, the measure combining information from ILI incidence by age group and influenza surveillance data by subtype is defined, for age group *g* and current month *t*, as:

$$flu_{i,g,t} = ILI_t * \frac{op_{g,t}}{Top_t} * \frac{pt_{i,g,t}}{Tpt_{g,t}}, \quad \text{(S4)}$$

where $pt_{i,t}$ is the numbers of specimens that tested positive for influenza subtype *i* (i = A-H1N1, A-H3N2, A-pH1N1, and B) in month *t*, and $Tpt_{g,t}$ the numbers of positive tests for all subtypes in age group g and month *t*.

For each age, we tried 216 models by combining nine different polynomial degrees (from the 2nd to the 10th), six orders of cyclical forms ($\frac{2\pi t}{12}$, …, $\frac{10\pi t}{12}$), and the two alternative influenza measures described above with and without their respective one-month lag term. We chose the model that provided the best fit according to AIC values, as we did for the Serfling model. Table S1 presents by single years of age the minimum AIC value obtained from each parametrization of influenza measures, the AIC change once the virus subtype information and the one-month lag variable are included, the statistical significance of this change, and the model providing the best fit. Alternatively, we applied a "*backward stepwise*" selection approach for each age (not shown here), starting with all flu activity terms in the model, removing at each step the least significant terms among the non-significant terms at the 5% level, and reintroducing, through re-estimation,

the most significant term among those that reach a significance level of 4% (for a more detailed description of the *backward stepwise* procedure, see Draper and Smith (1998)); the results obtained from this model selection strategy were not fundamentally different from those obtained from model selection based on AIC.

As shown in Table S1, there is considerable age variation with regard to the parameters that provided best AIC statistics. For example, neither the addition of one-month lag ILI terms nor the specification of virus subtype circulation (as in Eq. S4) provided significant improvement in model fit at age zero. We thus retained the default ILI model for that age, as we did generally until about age 20. From age 20 to age 65 approximately, the best models generally included terms specifying subtype circulation, usually without one-month lag terms for the younger portion of this age group (i.e., from age 20 to age 40), and then including these lag terms for the older portion (from age 40 to age 65). Interestingly, regarding the elderly (65+), models including lag terms systematically provided the best fit, while terms specifying subtype circulation were no longer kept in this age group.

Table S1 Fitting measures for alternative Surveillance-Serfling model parameterization and the model providing the best fit

Age	**Models with minimum AIC** for alternative influenza measures				**AIC Change**		**Model with Best Fit**
	**ILI**	**ILI + Lag**	**ILI by Subtype**	**ILI by Subtype + Lag**	**Subtype**	**Lag**	
**0**	1253.18	1249.56	1265.5	1265.92	12.32	-3.62	ILI
**1**	919.61	915.5	923.43	922.84	3.82	-4.11	ILI
**2**	725.45	723.27	731.26	738.15	5.81	-2.18	ILI
**3**	646.39	645.79	650.16	650.75	3.77	-0.6	ILI
**4**	573.62	574.81	579.92	583.57	6.3	1.19	ILI
**5**	559.41	561.4	558.08	564.56	-1.33	1.99	ILI
**6**	501.87	503.59	507.62	513.37	5.75	1.72	ILI
**7**	489.08	487.43	490.92	486.73	1.84	-1.65	ILI
**8**	455.09	454.21	458.14	463.76	3.05	-0.88	ILI
**9**	491.21	489.77	494.59	493.63	3.38	-1.44	ILI
**10**	466.71	470.62	470.56	478.43	3.85	3.91	ILI
**11**	453.68	450.11	458.19	455.83	4.51	-3.57	ILI
**12**	494.51	495.09	497.39	503.53	2.88	0.58	ILI
**13**	501.45	503.43	506.76	506.02	5.31	1.98	ILI
**14**	561.41	559.46	564.3	564.6	2.89	-1.95	ILI
**15**	517.14	510.13	519.99	516.18	2.85	-7.01*	ILI + Lag
**16**	513.91	515.85	519.42	526.81	5.51	1.94	ILI
**17**	581.24	569.74	584.72	579.01	3.48	-11.5*	ILI + Lag
**18**	634.97	627.68	637.26	635.55	2.29	-7.29*	ILI + Lag
**19**	620.07	622.02	621.99	624.73	1.92	1.95	ILI
**20**	709.84	708.75	710.87	711.1	1.03	-1.09	ILI
**21**	737.76	736.37	739.47	737.37	1.71	-1.39	ILI
**22**	756.61	753.29	756.38	746.25	-0.23	-3.32	ILI by Subtype + Lag
**23**	783.69	776.83	788.22	781.54	4.53	-6.86*	ILI + Lag
**24**	773.6	764.65	771.15	762.11	-2.45	-8.95*	ILI + Lag
**25**	772.79	763.86	761.31	752.58	-11.48*	-8.73*	ILI by Subtype + Lag
**26**	828.39	829.37	818.03	820.89	-10.36*	2.86	ILI by Subtype
**27**	822.74	823.57	816.73	819.48	-6.01*	2.75	ILI by Subtype
**28**	852.38	853.82	833.85	828.98	-18.53*	-4.87	ILI by Subtype
**29**	868.88	868.56	858.39	861.99	-10.49*	3.6	ILI by Subtype
**30**	868.75	864.05	840.67	838.92	-28.08*	-1.75	ILI by Subtype
**31**	898.65	897.54	888.72	892.16	-9.93*	3.44	ILI by Subtype
**32**	895.02	888.72	879.16	870.55	-15.86*	-8.61*	ILI by Subtype + Lag

Age	Models with minimum AIC for alternative influenza measures				AIC Change		Model with Best Fit
	ILI	ILI + Lag	ILI by Subtype	ILI by Subtype + Lag	Subtype	Lag	
**33**	902.35	902.82	879.92	885.36	-22.43*	5.44	ILI by Subtype
**34**	928.21	930.96	904.47	906.11	-23.74*	1.64	ILI by Subtype
**35**	957.63	950.01	930.15	920.91	-27.48*	-9.24*	ILI by Subtype + Lag
**36**	993.31	990.27	970.91	965.65	-22.4*	-5.26	ILI by Subtype
**37**	1003.80	1005.54	974.54	976.54	-29.26*	2.00	ILI by Subtype
**38**	1019.02	1019.71	994.78	997.54	-24.24*	2.76	ILI by Subtype
**39**	1039.89	1040.3	980.94	982.96	-58.95*	2.02	ILI by Subtype
**40**	1096.56	1096.6	1070.03	1071.26	-26.53*	1.23	ILI by Subtype
**41**	1117.01	1113.46	1070.26	1064.97	-46.75*	-5.29	ILI by Subtype
**42**	1139.82	1134.15	1098.46	1091.88	-41.36*	-6.58*	ILI by Subtype + Lag
**43**	1182.14	1178.64	1139.25	1129.00	-42.89*	-10.25*	ILI by Subtype + Lag
**44**	1174.09	1172.45	1139.65	1132.74	-34.44*	-6.91*	ILI by Subtype + Lag
**45**	1189.94	1173.68	1158.7	1137.30	-31.24*	-21.4*	ILI by Subtype + Lag
**46**	1193.53	1188.18	1162.46	1155.31	-31.07*	-7.15*	ILI by Subtype + Lag
**47**	1224.04	1220.95	1182.04	1176.25	-42.00*	-5.79	ILI by Subtype
**48**	1253.2	1241.69	1219.45	1200.79	-33.75*	-18.66*	ILI by Subtype + Lag
**49**	1230.24	1222.48	1195.82	1187.28	-34.42*	-8.54*	ILI by Subtype + Lag
**50**	1256.68	1239.82	1206.42	1178.99	-50.26*	-27.43*	ILI by Subtype + Lag
**51**	1278.99	1265.01	1257.43	1242.06	-21.56*	-15.37*	ILI by Subtype + Lag
**52**	1315.05	1310.33	1270.05	1264.2	-45.00*	-5.85	ILI by Subtype
**53**	1294.50	1291.12	1248.83	1241.36	-45.67*	-7.47*	ILI by Subtype + Lag
**54**	1348.10	1338.71	1322.77	1314.32	-25.33*	-8.45*	ILI by Subtype + Lag
**55**	1375.52	1365.52	1330.37	1310.74	-45.15*	-19.63*	ILI by Subtype + Lag
**56**	1340.04	1334.59	1305.5	1298.79	-34.54*	-6.71*	ILI by Subtype + Lag
**57**	1377.11	1360.76	1349.13	1320.86	-27.98*	-28.27*	ILI by Subtype + Lag
**58**	1393.18	1379.07	1363.76	1342.33	-29.42*	-21.43*	ILI by Subtype + Lag
**59**	1366.61	1358.58	1350.46	1343.21	-16.15*	-7.25*	ILI by Subtype + Lag
**60**	1400.24	1385.01	1385.27	1363.20	-14.97*	-22.07*	ILI by Subtype + Lag
**61**	1398.81	1382.72	1386.15	1364.82	-12.66*	-21.33*	ILI by Subtype + Lag
**62**	1406.38	1389.39	1401.95	1383.40	-4.43	-16.99*	ILI + Lag
**63**	1413.11	1387.61	1398.26	1371.30	-14.85*	-26.96*	ILI by Subtype + Lag
**64**	1411.28	1401.58	1407.53	1405.00	-3.75	-9.7*	ILI + Lag
**65**	1446.69	1439.4	1439.53	1434.19	-7.16*	-5.34	ILI + Lag
**66**	1420.72	1412.92	1422.14	1420.16	1.42	-7.80*	ILI + Lag
**67**	1478.71	1477.89	1469.73	1472.63	-8.98*	2.90	ILI by Subtype
**68**	1452.67	1443.32	1459.12	1451.76	6.45	-9.35*	ILI + Lag

Age	Models with minimum AIC for alternative influenza measures				AIC Change		Model with Best Fit
	ILI	ILI + Lag	ILI by Subtype	ILI by Subtype + Lag	Subtype	Lag	
**69**	1481.45	1470.96	1478.79	1465.77	-2.66	-10.49*	ILI + Lag
**70**	1514	1498.86	1520.66	1511.50	6.66	-15.14*	ILI + Lag
**71**	1528.38	1516.05	1520.66	1511.10	-7.72*	-9.56*	ILI + Lag
**72**	1542.65	1531.88	1550.73	1544.48	8.08	-10.77*	ILI + Lag
**73**	1581.03	1545.63	1593.46	1569.16	12.43	-35.4*	ILI + Lag
**74**	1625.8	1601.78	1630.62	1614.63	4.82	-24.02*	ILI + Lag
**75**	1610.39	1589.52	1612.54	1589.19	2.15	-20.87*	ILI + Lag
**76**	1669.03	1646.30	1688.18	1678.24	19.15	-22.73*	ILI + Lag
**77**	1655.57	1634.70	1672.49	1660.45	16.92	-20.87*	ILI + Lag
**78**	1687.91	1665.14	1698.20	1685.00	10.29	-22.77*	ILI + Lag
**79**	1671.13	1632.57	1686.63	1648.95	15.50	-38.56*	ILI + Lag
**80**	1741.59	1702.51	1751.36	1723.60	9.77	-39.08*	ILI + Lag
**81**	1756.79	1735.71	1762.31	1749.88	5.52	-21.08*	ILI + Lag
**82**	1796.94	1743.86	1808.05	1765.94	11.11	-53.08*	ILI + Lag
**83**	1818.83	1754.24	1817.96	1761.74	-0.87	-64.59*	ILI + Lag
**84**	1836.27	1791.06	1828.33	1798.58	-7.94*	-29.75*	ILI + Lag
**85**	1830.59	1766.90	1841.48	1798.47	10.89	-63.69*	ILI + Lag
**86**	1887.33	1830.52	1897.01	1858.01	9.68	-56.81*	ILI + Lag
**87**	1884.98	1845.15	1884.34	1863.21	-0.64	-39.83*	ILI + Lag
**88**	1868.12	1807.10	1878.52	1837.84	10.40	-61.02*	ILI + Lag
**89**	1895.92	1839.37	1890.94	1849.60	-4.98	-56.55*	ILI + Lag
**90**	1885.84	1833.45	1897.95	1872.23	12.11	-52.39*	ILI + Lag
**91**	1861.53	1818.42	1851.95	1821.22	-9.58*	-30.73*	ILI + Lag
**92**	1854.97	1819.36	1866.54	1851.63	11.57	-35.61*	ILI + Lag
**93**	1808.29	1756.82	1812.80	1784.57	4.51	-51.47*	ILI + Lag
**94**	1794.13	1759.20	1780.75	1759.90	-13.38*	-20.85*	ILI + Lag
**95**	1709.93	1682.89	1709.38	1698.82	-0.55	-27.04*	ILI + Lag
**96**	1655.25	1614.18	1657.18	1629.80	1.93	-41.07*	ILI + Lag
**97**	1582.43	1545.28	1593.54	1570.11	11.11	-37.15*	ILI + Lag
**98**	1538.01	1520.43	1541.58	1537.46	3.57	-17.58*	ILI + Lag
**99**	1468.42	1444.55	1465.76	1445.61	-2.66	-23.87*	ILI + Lag
**100**	1392.47	1373.31	1395.84	1388.01	3.37	-19.16*	ILI + Lag

* Statistically significant reduction in AIC values.

Note: The AIC is estimated to be -2LL+2k, where LL is the maximum log-likelihood and k is the number of parameters. A threshold value of 6 units is used to define whether the difference between two AIC statistic values is statistically significant, according to the selection criteria proposed by Hilbe (2011).

Specifying the Summer Season in the Serfling Model

Figure S1 presents estimates from the Serfling model using four different definitions of the summer period (i.e., May to September, May to October, June to September, and June to October) along with estimates from the Surveillance-Serfling model, while Fig. S2 shows the Lexis surfaces obtained from these models. Serfling estimates of death counts are generally sensitive to the definition of the summer period, with numbers yielded by those based on May to October or June to October being considerably lower compared to the others. For ages younger than 40, estimates obtained from the Surveillance-Serfling model are considerably lower and more erratic than those from any of the Serfling models (see Fig. S1). After that age, the surveillance model and the Serfling model based on the June to September summer period provides highly consistent estimates. Figure S1 shows that estimates from the Serfling model using June – September as baseline months fluctuate less over age, compared to other Serfling models; we thus retained months June – September to define the summer period in our final model (Fig. 4 in the manuscript, reproduced in color below as Fig. S3).

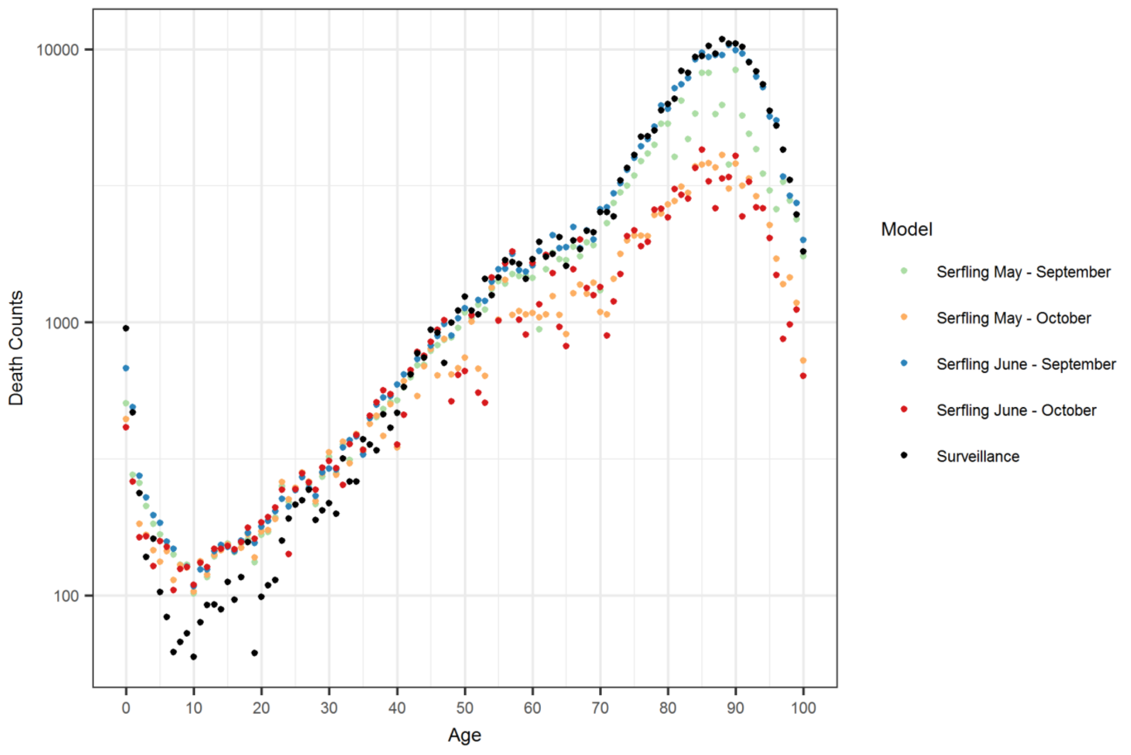

Fig. S1 Serfling and Surveillance-Serfling influenza death count estimates by age, between 1997 and 2016, according to alternative summer periods as baseline months

May – September ***May – October*** ***June – September*** ***June – October***

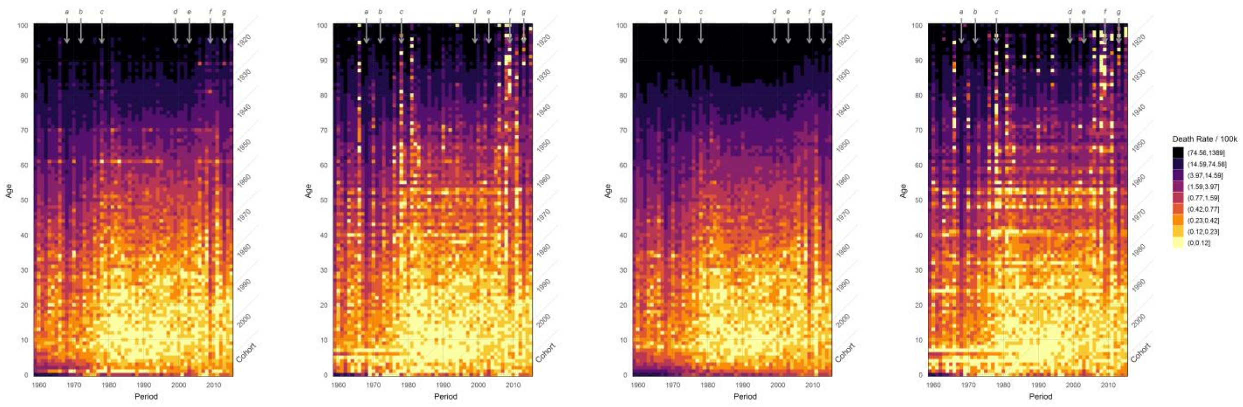

Fig. S2 Lexis surfaces from Serfling estimates, between 1997 and 2016, according to different definitions of the summer period

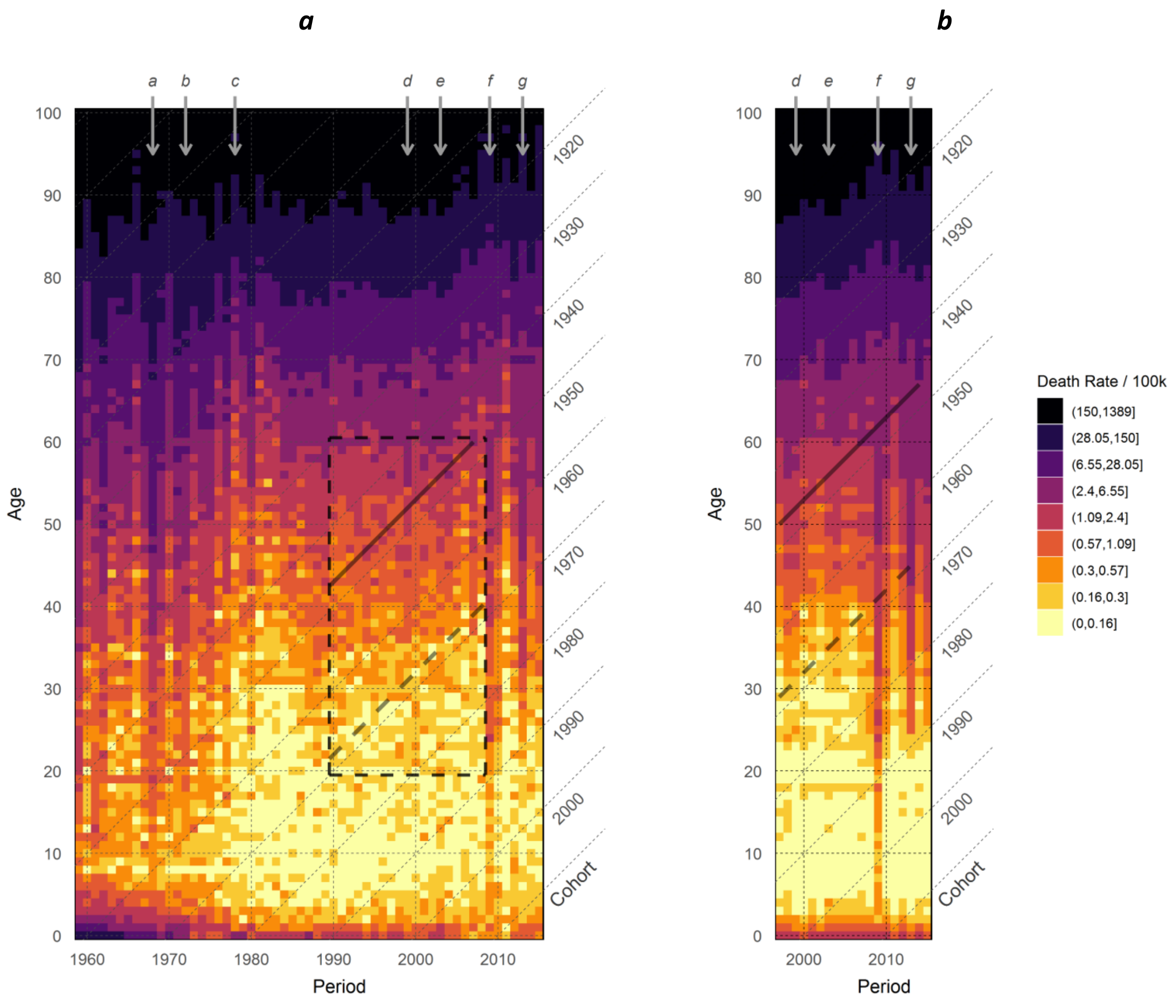

Fig. S3 Lexis surfaces of influenza mortality rates estimated by the Serfling model, 1959-2016 (a) and the Surveillance-Serfling model, 1997-2016 (b). The vertical arrows *a*, *b*, *d*, and e indicate periods of severe H3N2 epidemics. Arrow *c* marks the reappearance of H1N1 (1977-1978); arrows *f* and *g* indicate periods dominated by pH1N1. The solid and dashed black diagonal lines mark the 1947 and 1968 birth cohorts, respectively. The surface covered by the dashed square in Fig. 4a is shown in a three-dimensional perspective in Fig. 5 (note: this figure is the colored version of Fig.4 in the main manuscript)

Age-Period-Cohort Analysis

In this section, we present several descriptive steps and sensitivity analyses made to evaluate period- and cohort-based trends in influenza mortality estimates, obtained from the application of the Serfling model to the 1959-2016 period.

Before fitting any APC models, it is suggested to ascertain first whether the three-factor model describes data better than any simpler two-factor age-period (AP) or age-cohort (AC) model (Carstensen 2007; Clayton and Schifflers 1987; Holford 1991; Yang and Land 2013). Along with this evaluation, we also compared Poisson and Negative Binomial models to select the one that provides the best fit to our data. SE for our APC estimates were computed using a variance formula that accounts for autocorrelation (Hilbe 2011). The AICs presented in Table S2 suggest that the full APC model with Negative Binomial distribution for our response variable provides the best description of the data in terms of parsimony and goodness of fit.

Table S2 Akaike information criteria (AIC) values for APC models according to Poisson and negative binomial distributions

	k	Poisson		Negative Binomial	
		LL	AIC	LL	AIC
**A**	48	-98934	197964	-25736	51568
**AP**	75	-54503	109156	-23817	47784
**AC**	122	-67604	135452	-24609	49462
**APC**	148	-50074	100444	-23555	47406

Note: The AIC is estimated to be -2LL+2k, where LL is the maximum log-likelihood and k is the number of parameters.

Since the results in Table S2 indicate that the (three-factor) APC model accounts for significantly more variation than the simpler two-factor models, we proceeded to fit such a model, in which

the number of influenza-related deaths at age a and time t, i.e., $deaths_{a,t}$, which are expressed as follows:

$$log(deaths_{a,t}) = \theta_0 + \alpha_a + \beta_t + \gamma_c + log(exposure_{a,t}), \quad \text{(S5)}$$

where θ_0 is a constant, α_a the effect of age group a, β_t the effect of period t, γ_c the effect of cohort c, and $exposure_{a,t}$ the population of age a at risk at time t.

Given the perfect linear dependency among the age, period, and cohort components (Period – Age = Cohort), this model has an infinite number of solutions if no additional constraints are specified. Several alternatives have been proposed to address this so-called "identification problem," essentially by imposing external constraints that are either explicitly chosen by the researcher (e.g., the constraint-based models of Fienberg and Mason (1985)), or implicitly defined by the design matrix, which depends on the number of age groups and periods (and thus cohorts) included in the model itself (e.g., the ridge and intrinsic estimators of Fu (2000) and Yang et al., (2004)). Yet, these solutions are contentious because of the sensitivity of the outcomes to the constraint chosen, which validity can never be known with certainty (Clayton and Schifflers 1987; Fienberg 2013; Fosse and Winship 2018; Luo 2013; Tarone and Chu 1996). The main issue with all these models is indeed that they apportion the linear trend of change over time between period and cohort influences without providing a means to assess the validity of this decomposition using conventional statistical criteria (all solutions will yield the same goodness of fit statistics, e.g., the same AIC, BIC, Likelihood Ratio Test estimates, etc.). Hence, results obtained from this method should always be interpreted with caution and be seen as tentative or indicative rather than confirmatory.

The "long term slope" or "linear trend" that can be partitioned among period and cohort influences is known in the APC literature as the "drift parameter" (Carstensen 2007; Clayton and

Schifflers 1987; Holford 1991). To avoid confusion with *antigenic drift*, we prefer to use the terms *long-term slope* or *linear trend*. To analyze the contribution of period and cohort variations to the linear trend of influenza mortality over time, we chose to use the *APC-detrended* and the *Intrinsic Estimator* (IE) approaches to model mortality rates, which are described below.

Period- and Cohort-Detrended Models

According to Clayton and Schifflers (1987) Eq. S5 can be rewritten as a factor model:

$$log(deaths_{a,t}) = \theta_0 + \alpha_a + \beta_p^d + \delta_P(p - p_0) + \gamma_c^d + \delta_C(c - c_0) + log(exposure_{a,t}), \quad \text{(S6)}$$

where β_p^d and γ_c^d are the detrended period and cohort effects, δ_P and δ_C the linear trends of the period and cohort effects, p_0 and c_0 the reference period and cohort, respectively, and the remaining equation terms are defined as above. Thus, the overall linear trend of the model is

$$\delta = \delta_p + \delta_c \ . \quad \text{(S7)}$$

Note that, given the identification problem discussed above, the model yields the same fit for an infinite number of different partitions of the linear trend (δ) among the period (δ_p) and cohort (δ_c) linear trends.

Under the assumption that the long-term slope of mortality change can be entirely attributed to either period- or cohort-based factors, it is possible to estimate both period-detrended ($\delta_p = 0$) and cohort-detrended ($\delta_c = 0$) as alternative models, denoted here as APCd and ACPd, respectively. Different parameterization can be defined to extract the linear trend, either by using equal weight on all units in the dataset (Holford (1991)'s approach) or by using the death counts

or exposures as weights (Carstensen (2007)'s approach). Yet, the slopes of the linear trends obtained from these three approaches are very similar (-2.024%, -1.967%, and -1.978%, respectively) and the difference between them is not statistically significant at the 95% confidence level.

The Intrinsic Estimator

In order to address the identification problem arising from the perfect collinearity of the APC models, the Intrinsic Estimator (IE) method implicitly identifies a constraint that minimizes the APC parameter variance. The IE method can thus be seen in this regard as less arbitrary than other methods (Yang et al. 2004) since it does not leave the choice of the constraint to the researcher (note that in the case of the detrended method, one still has to choose to assign all the long-term slope to either cohort or period influences, which also amounts to the arbitrary addition of an external constraint). Yet, there remains controversy as to whether the IE method provides results that are truly less arbitrary (Luo 2013; Luo et al. 2016; Masters et al. 2014, 2016, 2018; Pelzer et al. 2015; te Grotenhuis et al. 2016; Xu and Powers 2016). In addition, a recent contribution by Fosse and Winship (2018) shows how the IE can be regrouped within a larger class of Moore-Penrose models and calls into question its applicability, at least based on extreme examples involving strongly "imbalanced" Lexis configurations, with as few as 5 age groups and up to 1,000 periods. That being said, we decided to include the IE estimates along the detrended estimates discussed above for comparison purposes, being aware of the limitations of all these methods. The results obtained from the APCd, APCd, and IE models are presented in Fig. 5, while the coefficients that were used to build this figure are presented at the end of this supplement (Table S5).

To demonstrate the applicability of the IE method to our data, we added sensitivity tests proposed in other studies (Luo et al. 2016; Masters et al. 2016, 2018; Yang and Land 2013). Note, however, that these tests, when successful, do not provide definitive support that the IE method could identify the “true” age, period, and cohort effects; as underlined above, there exists no unique, “best fit” solution to APC models.

We applied two different sensitivity tests for the IE. To test the robustness of the estimates and their sensitivity to model specification, we first changed the reference category from first to last of each of the age, period, and cohort terms of the model. Second, we used alternative numbers of years to define the cohorts and periods. Figure S4 shows that the three estimates are consistent, and do not substantively differ when changing the category of reference or the width of the age, period, and cohort intervals. Note that the purpose of the multi-year model (dashed line in Fig. S4) is uniquely to assess the sensitivity of the IE’s partition of first-order effects to alternative measurements of age, period, and cohort. Since cohort categories were generated as linearly dependent on the two-year age group and three-year time period (recall that Cohort = Period – Age), they do not correspond to actual cohorts, and thus, second-order cohort effects, discussed below, are not accurately obtained using this partition.

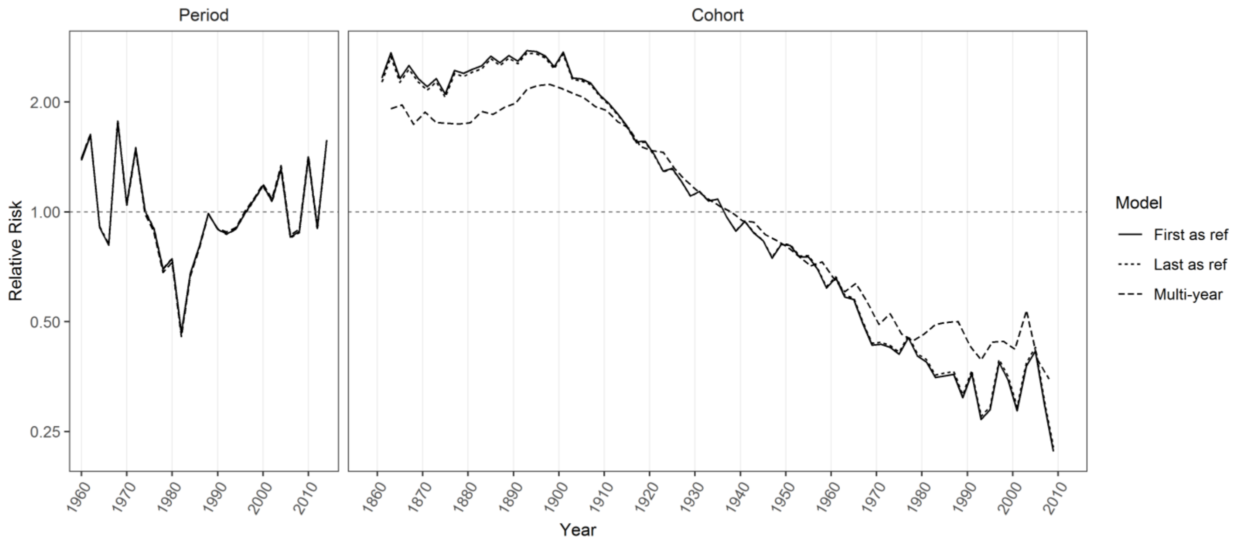

Fig. S4 Intrinsic estimates of period and cohort relative risks of influenza-related mortality, derived from the estimates of the Serfling model. The solid and dotted lines indicate, respectively, estimates from using the first and the last age, period, and cohort as reference, while the dashed line provides estimates obtained when using two-year periods and three-year age groups (hence labeled as "multi-year")

Changes in Trends

Unlike the above age, period and cohort trend estimates (first-order effects), which are dependent on the constraint imposed on the model, the changes in the direction of these trends (second-order effects) are invariant, whatever the constraint imposed, and thus unambiguously identifiable (Holford 1991; Keyes et al. 2010). Among these second-order effects, a *contrasts* approach allow us to identify "breakpoints" where period or cohort trends significantly change direction and to quantify the extent of these changes (O'Brien 2014; Shahpar and Li 1999; Tarone and Chu 1996). Thus, we are able to measure the difference between the slopes of two disjoint blocks composed of several consecutive periods or cohorts.

A contrast comparing slopes between two disjoint blocks of *n* consecutive period or cohort groups is defined as:

$$C = \pi_{k+n} - \pi_k - (\pi_{h+n} - \pi_h), \tag{S8}$$

where π_h and π_k are respectively the *h*-th and *k*-th period or cohort parameter estimates from any constraint-based model, with $h + n \leq k$.

Alternatively, by estimating the difference between the linear contrasts defined over the two blocks being compared, it is possible to account for the contribution of all periods or cohorts included within each block. For two disjoints blocks of four, five, six, and eight consecutive period or cohort groupings, the differences in the linear contrasts respectively follow the forms:

$$C_4 = 3\pi_{k+3} + \pi_{k+2} - \pi_{k+1} - 3\pi_k - (3\pi_{h+3} + \pi_{h+2} - \pi_{h+1} - 3\pi_h), \tag{S9}$$

$$C_5 = 2\pi_{k+4} + \pi_{k+3} - \pi_{k+1} - 2\pi_k - (2\pi_{h+4} + \pi_{h+3} - \pi_{h+1} - 2\pi_h), \tag{S10}$$

$$C_6 = 5\pi_{k+5} + 3\pi_{k+4} + \pi_{k+3} - \pi_{k+2} - 3\pi_{k+1} - 5\pi_k - (5\pi_{h+5} + 3\pi_{h+4} + \pi_{h+3} - \pi_{h+2} - 3\pi_{h+1} - 5\pi_h), \tag{S11}$$

$$C_8 = 7\pi_{k+7} + 5\pi_{k+6} + 3\pi_{k+5} + \pi_{k+4} - \pi_{k+3} - 3\pi_{k+2} - 5\pi_{k+1} - 7\pi_{k+7} - (7\pi_{h+7} + 5\pi_{h+6} + 3\pi_{h+5} + \pi_{h+4} - \pi_{h+3} - 3\pi_{h+2} - 5\pi_{h+1} - 7\pi_{h+7}). \tag{S12}$$

The SE of the contrast estimate is:

$$se = \sqrt{s' V_\pi s}, \tag{S13}$$

where s is the vector of coefficients defining the contrast (in Eqs. S8 to S12) and V_π is the variance-covariance matrix for the maximum likelihood estimates of the period or cohort effects.

In Fig. 8 and Table 2, the units of analysis correspond to two-year age, period, and cohort groupings (analyses using one-year groupings resulted in estimates that were merely unstable). To test the sensitivity of the contrasts presented in Table 2, we re-estimated the models using three-year instead of two-year APC groupings and the new contrast estimates are displayed in Table S3. Note that due to the change in the number of years in the age, period, and cohort groupings, some breakpoints are shifted right or left relative to those reported in Table 2. Overall, however, the results in Table S3 are remarkably similar to the results in Table 2.

Table S3 Contrasts in the linear trends between two disjoint blocks of three-year birth cohorts

#	Cohorts where changes in slope occur	Block 1	Block 2	Contrast a	Contrast b
1	~ 1896-1898	1881-1898	1896-1913	-0.402***	-0.979***
2	~ 1929-1931	1917-1931	1929-1943	0.166*	0.352*
3	~ 1944-1946	1932-1946	1944-1958	0.233**	0.524**
4	~ 1956-1958	1944-1958	1956-1970	-0.395***	-0.909***
5	~ 1968-1970	1956-1970	1968-1982	0.431**	0.943**
6	~ 1977-1985	1968-1979	1983-1994	-0.423**	-1.178*

Notes: Contrasts a is defined as the difference between the slopes formed by the straight lines connecting the first and the last trio of consecutive birth cohorts within each block. Contrast b is defined as the sum of differences of all slopes formed by any pair of cohorts taken in each block.
$^{+}$ $p < .10$; * $p < .05$; ** $p < .01$; *** $p < .001$.

Finally, in order to provide a broader comparative perspective on influenza mortality, we also conducted additional contrast analyses for all-cause mortality and for cardiovascular and respiratory diseases mortality, which are the major causes associated with death from influenza complications (Reichert et al. 2004; Simonsen et al. 2011). We used data from the *Human*

Mortality Database (2019) and from the *National Center for Health Statistics* (2018) over the period 1959-2016 to browse over the same years for which we have already identified significant contrasts (turning points) in influenza mortality. Changes in cohort mortality trends were also estimated two, four and 6 years *before and after* the identified turning points to assess the smoothness (or abruptness) of these changes (Table S4 presents contrasts up to four years before or after these turning points).

For example, the first estimated contrast on the first line of Table S4, i.e., -0.458, is the change in slope occurring in cohorts born in 1892-1897, i.e., four years before the cohorts born in 1896-1901, where the contrast for the cohort trend in influenza mortality is maximum (i.e., -0.528). The fact that all the contrasts located on this first line are all significant and of similar magnitude indicates that the change in slope for cohort born at the turn of the 20th century is rather smooth and not focussed on a specific year. As shown in Table S4, the other changes in slope in influenza mortality are usually "centered" in years with significant antigenic events, with much smaller, and not significant, contrasts in the previous or following four years. The largest changes for all-cause, respiratory-, and cardiovascular-related mortality, on the other hand, are more dispersed relative to the turning points identified for influenza.

Table S4 Contrasts in the linear trends between two disjoint blocks of two-year birth cohorts for deaths related to influenza, cardiovascular, and respiratory diseases, and for all-cause mortality

Cause	id	Cohorts	Contrast a					Contrast b				
			4 years before	2 years before	Centered	2 years after	4 years after	4 years before	2 years before	Centered	2 years after	4 years after
Influenza	1	~ 1896-1901	-0.458***	-0.393***	**-0.528*** **	-0.358***	-0.444***	-5.322***	-5.301***	**-5.583*** **	-4.620***	-5.208***
	2	~ 1928-1929	0.036	0.172*	**0.214***	0.071	-0.012	1.320+	1.760*	**1.801***	1.108	0.464
	3	~ 1946-1947	0.044	0.041	**0.246** **	0.032	-0.067	0.008	0.252	**0.774** **	0.178	-0.286
	4	~ 1956-1957	-0.173*	-0.157+	**-0.428*** **	-0.137	0.006	-0.384*	-0.576**	**-0.976*** **	-0.440+	-0.005
	5	~ 1968-1969	0.019	0.038	**0.392***	0.171	0.179	0.024	0.253	**0.837***	0.558	0.477
	6	~ 1976-1981	0.05	0.091	**-0.334***	-0.06	-0.278	0.109	0.070	**-0.587+**	-0.342	-0.697+
CVD[1]	1	~ 1896-1901	-0.142***	-0.127***	-0.152***	-0.128***	**-0.163*** **	-1.713***	-1.624***	-1.718***	-1.557***	**-1.760*** **
	2	~ 1928-1929	-0.024	-0.008	0.011	0.022	0.003	-0.127	-0.021	0.087	0.127	0.069
	3	~ 1946-1947	0.014	0.024	0.066***	**0.093*** **	0.074***	0.039	0.071	0.227***	**0.317*** **	0.247***
	4	~ 1956-1957	**0.102*** **	0.064***	0.029	0.008	0.030	**0.269*** **	0.159***	0.058	0.015	0.067+
	5	~ 1968-1969	0.033	0.064*	0.122***	**0.114*** **	0.075**	0.074	0.169**	0.300***	**0.309*** **	0.208***
	6	~ 1976-1981	0.000	-0.064*	-0.157***	**-0.223*** **	-0.201***	0.020	-0.158*	-0.399***	**-0.572*** **	-0.541***
RD[2]	1	~ 1896-1901	-0.058+	-0.089**	-0.155***	-0.155***	**-0.167*** **	-0.731*	-1.294***	-1.892***	-1.983***	**-2.071*** **
	2	~ 1928-1929	-0.158***	**-0.195*** **	-0.167***	-0.161***	-0.179***	-1.921***	**-2.265*** **	-2.225***	-2.117***	-2.071***
	3	~ 1946-1947	-0.023	-0.004	0.087**	**0.123*** **	0.067*	-0.096	-0.017	0.292***	**0.432*** **	0.230*
	4	~ 1956-1957	-0.135***	-0.157***	**-0.262*** **	-0.254***	-0.163***	-0.291***	-0.431***	-0.66***	**-0.654*** **	-0.444***
	5	~ 1968-1969	0.007	0.011	0.127**	**0.136** **	0.091+	-0.017	0.066	0.297***	**0.356*** **	0.275**
	6	~ 1976-1981	0.106*	0.073	0.017	-0.106+	**-0.175** **	0.274*	0.204+	0.043	-0.254+	**-0.467** **
All-Cause	1	~ 1896-1901	-0.015	-0.006	-0.041*	-0.029+	**-0.079*** **	-0.182	-0.172	-0.359*	-0.332*	**-0.725*** **
	2	~ 1928-1929	**-0.061*** **	-0.051**	-0.025	-0.002	0.016	**-0.621*** **	-0.533***	-0.373*	-0.195	0.083
	3	~ 1946-1947	0.066***	0.083***	**0.102*** **	0.081***	0.015	0.21***	0.279***	**0.348*** **	0.274***	0.054
	4	~ 1956-1957	-0.079***	-0.151***	**-0.207*** **	**-0.207*** **	-0.149***	-0.185***	-0.382***	-0.539***	**-0.541*** **	-0.38***
	5	~ 1968-1969	-0.051*	0.017	0.083***	**0.099*** **	0.076**	-0.138**	0.050	0.219***	**0.264*** **	0.198***
	6	~ 1976-1981	0.032	-0.031	-0.110***	-0.167***	**-0.212*** **	0.086	-0.100	-0.272***	-0.406***	**-0.537*** **

[1] Cardiovascular diseases.

[2] Respiratory diseases.

Notes: Contrast a is defined as the difference between the slopes formed by the straight lines connecting the first and the last pair of consecutive birth cohorts within each block. Contrast b is defined as the sum of differences of all slopes formed by any pair of cohorts taken in each block. The grey columns highlight the contrasts centered on cohorts listed in the third column (also in grey), i.e., for cohorts with the largest changes in slope in influenza mortality; values in red indicate the largest among the five contiguous contrasts, separately for contrasts a and b.

+p < .10; * p < .05; ** p < .01; *** p < .001.

Table S5 APCd, ACPd, and IE period and cohort effects on influenza-related mortality derived from the Serfling model, ages 5 to 100, 1959-1960 through 2014-2015 influenza seasons

Effect	Index	Years	APCd		ACPd		IE	
			Coefficient	SE	Coefficient	SE	Coefficient	SE
Period	1	1959-1960	0.933	0.037	0.382	0.037	0.404	0.037
	2	1961-1962	0.965	0.025	0.456	0.025	0.466	0.025
	3	1963-1964	0.344	0.025	-0.125	0.025	-0.105	0.025
	4	1965-1966	0.243	0.034	-0.185	0.034	-0.153	0.034
	5	1967-1968	0.972	0.031	0.585	0.031	0.587	0.031
	6	1969-1970	0.392	0.034	0.045	0.034	0.073	0.034
	7	1971-1972	0.656	0.021	0.350	0.021	0.357	0.021
	8	1973-1974	0.227	0.025	-0.038	0.025	-0.021	0.025
	9	1975-1976	0.150	0.035	-0.074	0.035	-0.062	0.035
	10	1977-1978	-0.146	0.037	-0.330	0.037	-0.303	0.037
	11	1979-1980	-0.127	0.028	-0.270	0.028	-0.285	0.028
	12	1981-1982	-0.657	0.030	-0.759	0.030	-0.743	0.03
	13	1983-1984	-0.31	0.028	-0.371	0.028	-0.387	0.028
	14	1985-1986	-0.218	0.023	-0.239	0.023	-0.248	0.023
	15	1987-1988	-0.046	0.021	-0.025	0.021	-0.045	0.021
	16	1989-1990	-0.175	0.025	-0.114	0.025	-0.131	0.025
	17	1991-1992	-0.276	0.020	-0.174	0.020	-0.188	0.020
	18	1993-1994	-0.275	0.022	-0.132	0.022	-0.150	0.022
	19	1995-1996	-0.235	0.021	-0.052	0.021	-0.068	0.021
	20	1997-1998	-0.147	0.024	0.077	0.024	0.050	0.024
	21	1999-2000	-0.128	0.023	0.137	0.023	0.119	0.023
	22	2001-2002	-0.253	0.024	0.052	0.024	0.032	0.024
	23	2003-2004	-0.090	0.021	0.257	0.021	0.231	0.021
	24	2005-2006	-0.578	0.026	-0.190	0.026	-0.190	0.026
	25	2007-2008	-0.557	0.031	-0.129	0.031	-0.120	0.031
	26	2009-2010	-0.089	0.046	0.380	0.046	0.412	0.046
	27	2011-2012	-0.558	0.037	-0.048	0.037	-0.053	0.037
	28	2013-2014	-0.017	0.044	0.533	0.044	0.520	0.044
Cohort	1	1860-1861	-0.733	0.121	0.776	0.121	0.697	0.121
	2	1862-1863	-0.506	0.085	0.962	0.085	0.909	0.085
	3	1864-1865	-0.576	0.085	0.851	0.085	0.782	0.085
	4	1866-1867	-0.461	0.079	0.926	0.079	0.865	0.079
	5	1868-1869	-0.500	0.074	0.846	0.074	0.792	0.074
	6	1870-1871	-0.492	0.071	0.814	0.071	0.76	0.071
	7	1872-1873	-0.417	0.054	0.847	0.054	0.789	0.054
	8	1874-1875	-0.470	0.055	0.753	0.055	0.704	0.055

Effect	Index	Years	APCd		ACPd		IE	
			Coefficient	SE	Coefficient	SE	Coefficient	SE
	9	1876-1877	-0.282	0.062	0.901	0.062	0.864	0.062
	10	1878-1879	-0.268	0.06	0.874	0.06	0.844	0.06
	11	1880-1881	-0.183	0.062	0.918	0.062	0.894	0.062
	12	1882-1883	-0.132	0.053	0.928	0.053	0.889	0.053
	13	1884-1885	-0.029	0.052	0.991	0.052	0.958	0.052
	14	1886-1887	0.003	0.059	0.982	0.059	0.955	0.059
	15	1888-1889	0.076	0.05	1.014	0.05	0.979	0.050
	16	1890-1891	0.091	0.05	0.989	0.05	0.961	0.050
	17	1892-1893	0.185	0.042	1.042	0.042	1.009	0.042
	18	1894-1895	0.211	0.039	1.026	0.039	0.994	0.039
	19	1896-1897	0.234	0.039	1.009	0.039	0.982	0.039
	20	1898-1899	0.211	0.039	0.945	0.039	0.923	0.039
	21	1900-1901	0.341	0.038	1.034	0.038	1.009	0.038
	22	1902-1903	0.216	0.036	0.868	0.036	0.846	0.036
	23	1904-1905	0.241	0.034	0.853	0.034	0.836	0.034
	24	1906-1907	0.250	0.033	0.821	0.033	0.803	0.033
	25	1908-1909	0.231	0.035	0.761	0.035	0.745	0.035
	26	1910-1911	0.215	0.034	0.704	0.034	0.688	0.034
	27	1912-1913	0.195	0.036	0.644	0.036	0.629	0.036
	28	1914-1915	0.176	0.039	0.584	0.039	0.569	0.039
	29	1916-1917	0.104	0.038	0.472	0.038	0.458	0.038
	30	1918-1919	0.141	0.037	0.467	0.037	0.456	0.037
	31	1920-1921	0.098	0.037	0.383	0.037	0.374	0.037
	32	1922-1923	0.045	0.039	0.289	0.039	0.280	0.039
	33	1924-1925	0.086	0.037	0.29	0.037	0.284	0.037
	34	1926-1927	0.037	0.037	0.200	0.037	0.195	0.037
	35	1928-1929	-0.007	0.041	0.116	0.041	0.114	0.041
	36	1930-1931	0.028	0.035	0.110	0.035	0.108	0.035
	37	1932-1933	0.053	0.042	0.094	0.042	0.098	0.042
	38	1934-1935	0.041	0.032	0.041	0.032	0.047	0.032
	39	1936-1937	-0.004	0.038	-0.045	0.038	-0.037	0.038
	40	1938-1939	-0.033	0.041	-0.115	0.041	-0.109	0.041
	41	1940-1941	0.027	0.035	-0.096	0.035	-0.084	0.035
	42	1942-1943	0.000	0.039	-0.163	0.039	-0.148	0.039
	43	1944-1945	0.008	0.038	-0.196	0.038	-0.186	0.038
	44	1946-1947	-0.062	0.043	-0.307	0.043	-0.291	0.043
	45	1948-1949	0.051	0.038	-0.235	0.038	-0.225	0.038
	46	1950-1951	0.086	0.038	-0.240	0.038	-0.222	0.038
	47	1952-1953	0.097	0.039	-0.270	0.039	-0.268	0.039

Effect	Index	Years	APCd		ACPd		IE	
			Coefficient	SE	Coefficient	SE	Coefficient	SE
	48	1954-1955	0.123	0.041	-0.285	0.041	-0.28	0.041
	49	1956-1957	0.099	0.043	-0.349	0.043	-0.348	0.043
	50	1958-1959	0.011	0.045	-0.478	0.045	-0.478	0.045
	51	1960-1961	0.122	0.045	-0.408	0.045	-0.403	0.045
	52	1962-1963	0.017	0.042	-0.554	0.042	-0.546	0.042
	53	1964-1965	0.013	0.045	-0.599	0.045	-0.569	0.045
	54	1966-1967	-0.056	0.052	-0.709	0.052	-0.688	0.052
	55	1968-1969	-0.126	0.062	-0.820	0.062	-0.790	0.062
	56	1970-1971	-0.063	0.062	-0.797	0.062	-0.783	0.062
	57	1972-1973	-0.067	0.063	-0.842	0.063	-0.807	0.063
	58	1974-1975	-0.082	0.061	-0.898	0.061	-0.843	0.061
	59	1976-1977	0.054	0.056	-0.802	0.056	-0.775	0.056
	60	1978-1979	0.019	0.067	-0.879	0.067	-0.856	0.067
	61	1980-1981	0.069	0.080	-0.869	0.08	-0.869	0.08
	62	1982-1983	0.016	0.077	-0.962	0.077	-0.952	0.077
	63	1984-1985	0.025	0.082	-0.995	0.082	-0.978	0.082
	64	1986-1987	0.106	0.094	-0.955	0.094	-0.933	0.094
	65	1988-1989	-0.092	0.076	-1.193	0.076	-1.143	0.076
	66	1990-1991	0.073	0.070	-1.069	0.070	-1.017	0.070
	67	1992-1993	-0.132	0.089	-1.315	0.089	-1.248	0.089
	68	1994-1995	-0.035	0.092	-1.258	0.092	-1.193	0.092
	69	1996-1997	0.251	0.078	-1.014	0.078	-0.971	0.078
	70	1998-1999	0.165	0.104	-1.14	0.104	-1.057	0.104
	71	2000-2001	0.018	0.112	-1.328	0.112	-1.263	0.112
	72	2002-2003	0.303	0.135	-1.084	0.135	-1.025	0.135
	73	2004-2005	0.481	0.138	-0.946	0.138	-0.911	0.138
	74	2006-2007	0.235	0.172	-1.233	0.172	-1.168	0.172
	75	2008-2009	-0.171	0.138	-1.680	0.138	-1.622	0.138

References

Carstensen, B. (2007). Age-period-cohort models for the Lexis diagram. *Statistics in Medicine*, *26*(15), 3018–3045. doi:10.1002/sim.2764

Clayton, D., & Schifflers, E. (1987). Models for temporal variation in cancer rates. II: Age–period–cohort models. *Statistics in Medicine, 6*(4), 469–481. doi:10.1002/sim.4780060406

Draper, N. R., & Smith, H. (1998). *Applied Regression Analysis* (3. edition.). New York: Wiley-Interscience.

Dushoff, J., Plotkin, J. B., Viboud, C., Earn, D. J. D., & Simonsen, L. (2006). Mortality due to influenza in the United States--an annualized regression approach using multiple-cause mortality data. *American Journal of Epidemiology*, *163*(2), 181–187. doi:10.1093/aje/kwj024

Fienberg, S. E. (2013). Cohort Analysis' Unholy Quest: A Discussion. *Demography*, *50*(6), 1981–1984. doi:10.1007/s13524-013-0251-z

Fienberg, S. E., & Mason, W. M. (1985). Specification and Implementation of Age, Period and Cohort Models. In *Cohort Analysis in Social Research* (pp. 45–88). Springer, New York, NY. doi:10.1007/978-1-4613-8536-3_3

Fosse, E., & Winship, C. (2018). Moore–Penrose Estimators of Age–Period–Cohort Effects: Their Interrelationship and Properties. *Sociological Science*, *5*(14), 304--334. doi:10.15195/v5.a14

Fu, W. J. (2000). Ridge estimator in singulah oesiun with application to age-period-cohort analysis of disease rates. *Communications in Statistics - Theory and Methods*, *29*(2), 263–278. doi:10.1080/03610920008832483

Hilbe, J. M. (2011). *Negative Binomial Regression* (2 edition.). Cambridge, UK ; New York: Cambridge University Press.

HMD. (2019). Human Mortality Database. University of California, Berkeley (USA), and Max Planck Institute for Demographic Research (Germany). http://www.mortality.org/. Accessed 15 March 2019

Holford, T. R. (1991). Understanding the effects of age, period, and cohort on incidence and mortality rates. *Annual Review of Public Health*, *12*, 425–457. doi:10.1146/annurev.pu.12.050191.002233

Keyes, K. M., Utz, R. L., Robinson, W., & Li, G. (2010). What is a cohort effect? Comparison of three statistical methods for modeling cohort effects in obesity prevalence in the United States, 1971–2006. *Social science & medicine (1982)*, *70*(7), 1100–1108. doi:10.1016/j.socscimed.2009.12.018

Lemaitre, M., Carrat, F., Rey, G., Miller, M., Simonsen, L., & Viboud, C. (2012). Mortality Burden of the 2009 A/H1N1 Influenza Pandemic in France: Comparison to Seasonal Influenza and the A/H3N2 Pandemic. *PLoS ONE*, *7*(9). doi:10.1371/journal.pone.0045051

Luo, L. (2013). Assessing Validity and Application Scope of the Intrinsic Estimator Approach to the Age-Period-Cohort Problem. *Demography*, *50*(6), 1945–1967. doi:10.1007/s13524-013-0243-z

Luo, L., Hodges, J., Winship, C., & Powers, D. (2016). The Sensitivity of the Intrinsic Estimator to Coding Schemes: Comment on Yang, Schulhofer-Wohl, Fu, and Land. *American Journal of Sociology*, *122*(3), 930–961. doi:10.1086/689830

Masters, R. K., Hummer, R. A., Powers, D. A., Beck, A., Lin, S.-F., & Finch, B. K. (2014). Long-Term Trends in Adult Mortality for U.S. Blacks and Whites: An Examination of Period- and Cohort-Based Changes. *Demography*, *51*(6), 2047–2073. doi:10.1007/s13524-014-0343-4

Masters, R. K., Powers, D. A., Hummer, R. A., Beck, A., Lin, S.-F., & Finch, B. K. (2016). Fitting Age-Period-Cohort Models Using the Intrinsic Estimator: Assumptions and Misapplications. *Demography*, *53*(4), 1253–1259. doi:10.1007/s13524-016-0481-y

Masters, R. K., Tilstra, A. M., & Simon, D. H. (2018). Explaining recent mortality trends among younger and middle-aged White Americans. *International Journal of Epidemiology*, *47*(1), 81–88. doi:10.1093/ije/dyx127

NCHS. (2018). Data Access - Vital Statistics Online. http://www.cdc.gov/nchs/data_access/Vitalstatsonline.htm. Accessed 5 December 2018

Nguyen, A. M., & Noymer, A. (2013). Influenza Mortality in the United States, 2009 Pandemic: Burden, Timing and Age Distribution. *PLoS ONE*, *8*(5), e64198. doi:10.1371/journal.pone.0064198

O'Brien, R. M. (2014). Estimable functions in age-period-cohort models: a unified approach. *Quality & Quantity*, *48*(1), 457–474. doi:10.1007/s11135-012-9780-6

Pelzer, B., te Grotenhuis, M., Eisinga, R., & Schmidt-Catran, A. W. (2015). The Non-uniqueness Property of the Intrinsic Estimator in APC Models. *Demography*, *52*(1), 315–327. doi:10.1007/s13524-014-0360-3

Reichert, T. A., Simonsen, L., Sharma, A., Pardo, S. A., Fedson, D. S., & Miller, M. A. (2004). Influenza and the Winter Increase in Mortality in the United States, 1959–1999. *American Journal of Epidemiology*, *160*(5), 492–502. doi:10.1093/aje/kwh227

Serfling, R. E. (1963). Methods for current statistical analysis of excess pneumonia-influenza deaths. *Public Health Reports*, *78*(6), 494–506.

Shahpar, C., & Li, G. (1999). Homicide Mortality in the United States, 1935–1994: Age, Period, and Cohort Effects. *American Journal of Epidemiology*, *150*(11), 1213–1222. doi:10.1093/oxfordjournals.aje.a009948

Simonsen, L., Reichert, T. A., Viboud, C., Blackwelder, W. C., Taylor, R. J., & Miller, M. A. (2005). Impact of influenza vaccination on seasonal mortality in the US elderly population. *Archives of Internal Medicine*, *165*(3), 265–272. doi:10.1001/archinte.165.3.265

Simonsen, L., Viboud, C., Taylor, R. J., & Miller, M. A. (2011). The Epidemiology of Influenza and Its Control. In R. Rappuoli & G. D. Giudice (Eds.), *Influenza Vaccines for the Future* (pp. 27–54). Springer Basel. doi:10.1007/978-3-0346-0279-2_2

Tarone, R., & Chu, K. C. (1996). Evaluation of birth cohort patterns in population disease rates. - PubMed - NCBI. *American Journal of Epidemiology*, *143*(1), 85–91.

te Grotenhuis, M., Pelzer, B., Luo, L., & Schmidt-Catran, A. W. (2016). The Intrinsic Estimator, Alternative Estimates, and Predictions of Mortality Trends: A Comment on Masters, Hummer, Powers, Beck, Lin, and Finch. *Demography*, *53*(4), 1245–1252. doi:10.1007/s13524-016-0476-8

Thompson, W. W., Weintraub, E., Dhankhar, P., Cheng, P.-Y., Brammer, L., Meltzer, M. I., et al. (2009). Estimates of US influenza-associated deaths made using four different methods. *Influenza and other respiratory viruses*, *3*(1), 37–49. doi:10.1111/j.1750-2659.2009.00073.x

Xu, M., & Powers, D. A. (2016). Bayesian Ridge Estimation of Age-Period-Cohort Models. In R. Schoen (Ed.), *Dynamic Demographic Analysis* (pp. 337–359). Springer International Publishing. doi:10.1007/978-3-319-26603-9_17

Yang, Y., Fu, W. J., & Land, K. C. (2004). A Methodological Comparison of Age-Period-Cohort Models: The Intrinsic Estimator and Conventional Generalized Linear Models. *Sociological Methodology*, *34*(1), 75–110. doi:10.1111/j.0081-1750.2004.00148.x

Yang, Y., & Land, K. C. (2013). *Age-Period-Cohort Analysis: New Models, Methods, and Empirical Applications*. Boca Raton, FL: Chapman and Hall/CRC.
